# A Review on the Design Strategies of Copper-Based Catalysts for Enhanced Activity and Stability in Methanol Reforming to Hydrogen

**DOI:** 10.3390/nano15141118

**Published:** 2025-07-18

**Authors:** Shuang Pang, Xueying Dou, Wei Zhao, Suli Bai, Bo Wan, Tiaoxia Wang, Jing-He Yang

**Affiliations:** 1 Hami Vocational and Technical College, Hami 839000, China; PangS100936@163.com (S.P.); chocolatedoushabao@163.com (X.D.); zwzw8686@outlook.com (W.Z.); djchouchou_1010@163.com (S.B.); annywan_hm@163.com (B.W.); WTX204806@outlook.com (T.W.); 2 School of Chemical Engineering, Zhengzhou University, Zhengzhou 450001, China

**Keywords:** copper-based catalysts, methanol reforming to hydrogen, design strategy

## Abstract

Methanol Steam Reforming (MSR) is one of the most promising technologies in the hydrogen economy, and copper-based catalysts have become the core materials in this field due to their high activity and low cost. In this paper, we systematically review the design strategies of copper-based catalysts in MSR reactions in recent years, including structure control, component optimization, support effect, and surface modification. We focus on the mechanisms of active site exposure, improvement of anti-sintering ability, and the enhancement of anti-carbon deposition performance. Finally, we summarize the challenges of current research and propose the future development direction. This review aims to provide a reference for subsequent related research through the experience of this paper.

## 1. Introduction

In the context of the transition of the global energy structure to low-carbon, hydrogen energy has become the core of the strategic layout of various countries due to its high energy density and zero carbon emission characteristics [1,2]. The International Energy Agency predicts that hydrogen will account for 13% of global energy consumption by 2050, with green and blue hydrogen being the main development directions [3]. However, the large-scale production of green hydrogen is limited by the high energy consumption and infrastructure bottlenecks of water electrolysis technology, while blue hydrogen relies on the efficient operation of carbon capture and storage technology [4]. In this context, the distributed hydrogen production technology based on liquid organic hydrogen storage carriers stands out due to the convenience and safety of raw material storage and transportation [5]. Methanol steam reforming for hydrogen production is an ideal choice for small- and medium-scale hydrogen production scenarios due to its advantages of mild reaction conditions, high hydrogen purity of the product, wide range of methanol sources and low liquid storage and transportation costs [6]. However, the commercial application of this technology still faces the challenge of insufficient performance of the core catalyst—the specific surface area of the copper-based active components is easy to decrease due to sintering in the high-temperature reaction, the carbon deposition generated by the side reaction covers the active site, and the poisoning of trace sulfur and chlorine impurities in the raw material jointly leads to the rapid deactivation of the catalyst. The catalyst deactivation rate in industrial-grade reactors can reach 5–10% per 1000 h, significantly increasing system maintenance and feedstock costs [7].

In view of the above bottlenecks, in recent years researchers have systematically optimized the active site exposure capacity, sintering resistance and carbon deposition resistance of copper-based catalysts through multi-dimensional strategies such as nanostructure design, support interface engineering, and alloying modification [8]. The purpose of this paper is to sort out the design logic and performance optimization mechanism of copper-based catalysts in methanol steam reforming reactions, and to discuss the key challenges and future directions in the process of industrialization, so as to provide theoretical support for the development of efficient and stable hydrogen production catalysts [9]. The activity and stability of copper-based catalysts are essentially regulated by the synergistic effect between the intrinsic properties of the active site and the reaction pathway, and the dynamic coupling of reaction conditions and operating parameters will further change the surface adsorption behavior and mass transfer process [10]. For example, although the temperature increase can accelerate the kinetics of methanol dehydrogenation, the particle mobility increases dramatically and the sintering rate increases exponentially after the Tammann temperature of copper is exceeded. Fluctuations in raw material composition (e.g., formic acid impurities dehydrating to form dimethyl ether) can occupy the active site, resulting in a 20–30% reduction in initial conversion rate [11]. This complex coupling effect requires that the catalyst design must take into account the synergistic control of thermodynamic stability and surface chemistry.

The core of the failure mechanism of catalysts lies in the physical coverage of the active site and the destruction of the chemical structure. Sintering results from the high-temperature surface migration of copper particles and the ripening process of Ostwald, and the experimental results show that when the temperature exceeds 407 °C, the copper atoms are diffused and aggregated into large-sized particles through the surface of the carrier, which greatly reduces the density of the active site [12]. Carbon deposition deactivation involves the deposition of secondary reactive carbon species, including graphitic carbon formation via the Boudouard reaction and the direct decomposition of methanol to form amorphous carbon, and the carbon layer not only blocks the carrier pores, but also hinders the mass transfer of reactants. Acidic carriers such as alumina can exacerbate carbon deposition by promoting methanol dehydration, while alkaline additives can neutralize acidic sites, reducing the rate of carbon deposition by 40–60% [13]. Chemical poisoning is mainly due to the irreversible adsorption of impurities, for example, 1 ppm hydrogen sulfide, forming Cu-S bonds with the copper surface to block methanol adsorption. It should be emphasized that the three deactivation mechanisms often work synergistically under actual working conditions: particle coarsening caused by sintering exposes the less-reactive crystal plane, which intensifies carbon monoxide adsorption and carbon deposition. The adiabatic effect of the carbon layer locally raises the temperature and further accelerates the sintering process.

Therefore, the rational design of copper-based catalysts needs to analyze the dynamic evolution of active sites at the atomic scale. The results show that the methanol steam reforming reaction is mainly completed in series by methanol dehydrogenation and the water-gas shift, and its path is regulated by the chemical state and surface structure of copper species. The active center is dominated by copper metal and copper oxide, the former leading the dehydrogenation of methanol, and the latter promoting the water–gas shift reaction by enhancing the adsorption and dissociation ability of water molecules, and reducing the concentration of carbon monoxide in the product. The size effect of nanoparticles is also crucial: when the copper particle size is less than 5 nm, the proportion of low-coordination atoms on the surface increases, which is conducive to breaking the methanol-carbon-oxygen bond. However, particles that are too small will cause excessive adsorption of carbon monoxide due to changes in electronic structure, which will induce side reactions of carbon deposition. Recent studies have also revealed that the oxygen vacancies at the copper-cerium oxide interface can accelerate hydroxyl group migration and promote carbon monoxide oxidation kinetics. However, the relative contribution of copper metal to copper oxide is still controversial, and some theoretical calculations suggest that copper oxide sites may dominate the methanol conversion rate by decreasing the desorption energy barrier of methoxy intermediates. This controversy highlights the complexity of the dynamic evolution of active sites, which needs to be further elucidated in combination with in situ characterization techniques.

## 2. Key Influencing Factors of Copper-Based Catalyst Activity and Stability

### 2.1. Active Site and Reaction Mechanism

**Figure 1:**
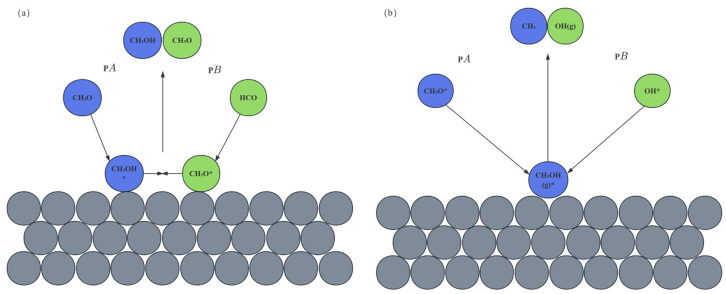
Schematic representation of the L-H mechanism (**a**) and the E-R mechanism (**b**).

The catalytic performance of MSR is highly dependent on the synergistic effect between the intrinsic properties of the active sites and the reaction pathway in the copper-based catalyst [14]. The results show that the MSR reaction is mainly completed in series by two key steps: methanol dehydrogenation and water–gas transformation, and its reaction path is regulated by the chemical state and surface structure of copper species [15,16]. The active centers of copper-based catalysts are mainly metallic copper (Cu^0^) and oxidized copper (Cu^+^), of which Cu^0^ is considered to be the main active site for methanol dehydrogenation, while Cu^+^ promotes the water–gas shift reaction (WGSR) by enhancing the adsorption and dissociation ability of H_2_O, thereby reducing the CO concentration in the products (as shown in Figure 1) [17,18,19]. For example, in situ X-ray absorption fine structure spectroscopy (XAFS) analysis showed that the presence of ZnO in Cu/ZnO/Al_2_O_3_ catalyst could stabilize Cu^+^ species and form Cu–O–Zn interface sites, significantly improving CO_2_ selectivity [20]. Additionally, the size effect of copper nanoparticles cannot be ignored: when the Cu particle size is less than 5 nm, the proportion of low-coordination atoms on the surface increases, which is conducive to cleaving C-O bonds in methanol molecules. However, particles that are too small may lead to the excessive adsorption of CO due to changes in electronic structure, which may induce side reactions of carbon deposition [21]. In recent years, some studies have also proposed that the oxygen vacancy at the Cu–CeO_2_ interface can promote the rapid migration of hydroxyl groups (-OH) and accelerate the dynamic process of CO oxidation to CO_2_ [22]. However, there is still controversy about the relative contribution of Cu^0^ to Cu^+^. For example, some scholars have pointed out that the Cu^+^ site may dominate the methanol conversion rate by decreasing the desorption energy barrier of CH_3_O* intermediates through density functional theory (DFT) calculations [23]. DFT calculations show that the adsorption energy of methanol by low-coordination Cu atoms (e.g., stepped edge sites) is significantly higher than that on planar sites (−0.52 eV), and the C-O bond cleavage barrier (0.8 eV) in the Cu(110) surfaces is 33% lower than that on Cu(111), which explains the highly active nature of small-sized particles. This controversy highlights the complexity of the dynamic evolution of active sites, which needs to be further clarified in conjunction with operando characterization techniques.

In order to verify under which specific reaction conditions (e.g., water/methanol ratio, temperature) Cu or CuO are dominant, a series of reviews of the specific reaction conditions are as follows:

(1) Influence of temperature: At lower temperatures (<250 °C), Cu is more stable and plays a key role in the water–gas shift reaction (WGSR) to promote the conversion of CO to CO_2_. At higher temperatures (>300 °C), CuO is more dominant and dominates the methanol dehydrogenation reaction. For example, studies have shown that at 200 °C, the proportion of Cu is as high as 60%, and at 350 °C the proportion of CuO increases to 85% [24].
(2) Effect of water/methanol ratio: under the high water/methanol ratio (>3:1), H_2_O dissociation was enhanced, and the Cu ratio increased, which promoted WGSR to reduce CO concentration. It has been found that when the water/methanol ratio is increased from 1:1 to 4:1, the Cu ratio increases from 30% to 50%, and the CO selectivity decreases to <1% [25].
(3) Effect of support: ZnO support stabilizes Cu by forming Cu–O–Zn interface, and some studies have shown that the proportion of Cu in Cu/ZnO catalyst is 25% higher than that of Cu/Al_2_O_3_ [26].

These findings collectively underscore the dynamic interplay between reaction conditions and the prevailing copper species (Cu^0^ vs. Cu^+^) in governing the MSR reaction pathways. However, achieving optimal catalytic performance necessitates not only understanding this intrinsic copper chemistry but also strategically modulating it through extrinsic factors. This is where the critical role of catalyst supports and promoters comes into play. The selection of an appropriate support material fundamentally alters the microenvironment surrounding the copper nanoparticles, profoundly influencing their chemical state, dispersion, stability, and ultimately, the synergistic execution of the methanol dehydrogenation and WGSR steps.

Beyond the intrinsic properties of the copper species themselves, the selection of appropriate promoters/supports plays a crucial role in modulating the chemical state, dispersion, and stability of the active sites, thereby significantly influencing the overall catalytic performance in MSR. As summarized in Table 1, different supports exert distinct effects:

### 2.2. Deactivation Mechanism

The deactivation of copper-based catalysts in MSR reactions is mainly caused by three mechanisms: sintering, carbon deposition, and chemical poisoning [32]. These mechanisms can be attributed to the physical coverage of the active site or the destruction of the chemical structure. Sintering deactivation is due to the surface migration of copper particles at high temperatures and the Ostwald curing process. Experiments show that when the reaction temperature exceeds the Tammann temperature of Cu^0^ (about 407 °C), Cu^0^ atoms diffuse through the support surface and aggregate into large-sized particles, resulting in a decrease in specific surface area and a sharp decrease in the density of the active site [33,34].

**Figure 2:**
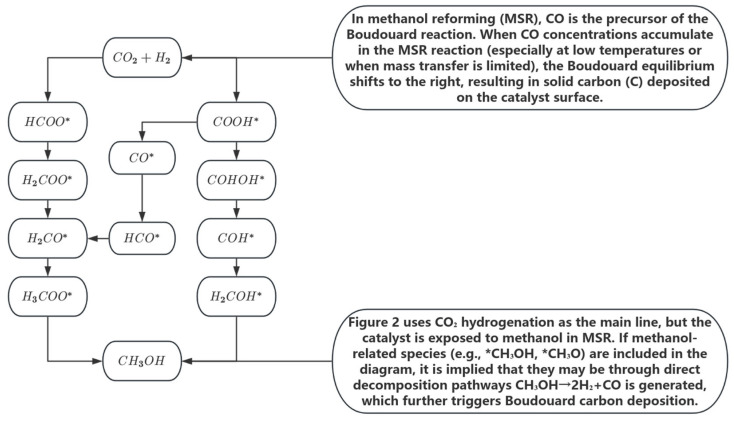
Reaction mechanism of CO_2_ hydrogenation to methanol.

For example, after running the unmodified Cu/Al_2_O_3_ catalyst at 300 °C for 100 h, the average diameter of Cu particles increased from 4.2 nm to 15.6 nm, and the methanol conversion attenuation rate reached 62% [35,36]. Carbon deposition deactivation is related to the deposition of carbon species on the surface of the catalyst by side reactions, which mainly include two pathways: one is the Boudouard reaction (2CO → C + CO_2_) to generate graphitic carbon, and the other is the amorphous carbon produced by direct methanol decomposition (CH_3_OH → 2H_2_ + CO) (as shown in Figure 2) [35]. The sintering deactivation is due to Ostwald curation: at a temperature of >407 °C, Cu atoms diffuse across the carrier surface (activation energy ≈ 120 kJ/mol), resulting in a rightward shift in the particle size distribution. Carbon deposition involves two pathways: (1) Boudouard equilibrium reaction (2CO ⇌ C + CO_2_, ΔG_{300 °C} = −120 kJ/mol); (2) Methanol directly decomposes to amorphous carbon. Acidic carriers (e.g., γ-Al_2_O_3_) promote the dehydration of methanol to dimethyl ether (DME) through the B acid site, and its strong adsorption covers the active site. Taking Cu/ZnO/Al_2_O_3_ as an example, the alkaline MgO additives can neutralize the acid position and reduce the carbon deposition rate by 60%. Experimental data further quantify this synergistic effect: when the carbon deposition coverage reaches 8 wt%, in situ thermography shows a 50–80 °C increase in the local temperature of the catalyst due to the adiabatic effect, resulting in a 3–5-fold increase in the sintering rate of copper particles (from 0.02 nm/min to 0.06–0.10 nm/min). For every 1 μm increase in carbon deposition thickness, the local temperature rises by about 35 °C, and the sintering activation energy decreases by 15 kJ/mol. The model analysis shows that for every 1 wt% increase in carbon deposition, the sintering rate constant increases by 12% on average.

The carbon layer not only covers the active site, but also clogs the carrier pores and hinders mass transfer of reactants. Studies have shown that acidic carriers, such as Al_2_O_3_, can indirectly exacerbate carbon deposition by promoting the dehydration of methanol to dimethyl ether (DME) [37]. The introduction of alkaline additives neutralizes acidic sites and reduces the rate of carbon deposition by 40–60% [38]. Chemical poisoning mainly stems from the irreversible adsorption of impurities in raw materials at the active site. For example, even at concentrations as low as 1 ppm, H_2_S can form Cu-S bonds with the Cu^0^ surface to block methanol adsorption [39]. The Cl^−^ ion destroys the Cu–ZnO interface structure, resulting in the stripping of active components. It is worth noting that the three deactivation mechanisms often work synergistically under actual working conditions: particle coarsening caused by sintering exposes more low-reactive crystal planes, which aggravates CO adsorption and carbon deposition [40]. The thermal insulation effect of the carbon deposit layer locally increases the catalyst temperature and further accelerates sintering [41]. Therefore, the anti-deactivation strategy needs to take into account the synergistic regulation of thermodynamic stability and surface chemical properties.

The deactivation of copper-based catalysts in methanol steam reforming reaction is mainly caused by the synergistic effect of sintering, carbon deposition and chemical poisoning, which is essentially the physical coverage of the active site or the destruction of the chemical structure, and the selection of support materials plays a decisive role. First of all, the sintering deactivation results from the surface migration of copper particles at high temperatures and the Ostwald curing process. When the temperature exceeds the Tamman temperature of Cu^0^ (about 407 °C), Cu^0^ atoms are easily diffused and aggregated into large particles, resulting in a sharp decrease in the density of the active site. The physicochemical properties of the support directly determine its ability to resist sintering: weak interaction carriers (such as unmodified γ-Al_2_O_3_) have limited resistance to sintering (e.g., Cu/Al_2_O_3_ is operated at 300 °C for 100 h, and the particle size increases significantly, and the conversion rate decays by 62%) [30]. The strong metal–support interaction (SMSI) support (e.g., ZnO, CeO_2_) can improve the stability through anchoring. The most effective strategy is a physical confinement (e.g., SiO_2_ shell encapsulation or zeolite pore limiting) that can significantly reduce the sintering rate (e.g., Cu particles in the SiO_2_ confinement only grow by 1.4 nm in 300 h at 300 °C) [31]. Second, carbon deposition deactivation is mainly caused by the deposition of carbon species resulting from the Boudouard reaction (2CO ⇌ C + CO_2_) and direct methanol decomposition (CH_3_OH → 2H_2_ + CO), covering the active site and blocking the pores. The chemical properties of the support surface (especially the acidity and alkalinity) are the key factors affecting carbon deposition: acidic supports (such as γ-Al_2_O_3_) strongly promote the dehydration of methanol to dimethyl ether (DME) due to their Bronster acid position (B acid position), which is an important carbon deposit precursor, which will significantly aggravate coking. Conversely, basic additives (e.g., MgO) or neutral/basic carriers (e.g., modified CeO_2_/ZrO_2_) can effectively neutralize acidic sites and reduce the rate of carbon deposition by 40–60% (e.g., Cu-ZnO-MgO systems inhibit carbon deposition through acid-base synergy) [28]. In addition, the oxygen vacancies of redox carriers (e.g., CeO_2_) can actively oxidize carbon species or their precursors (e.g., CO), and their density is positively correlated with carbon deposition resistance. Optimizing pore structures (e.g., MOFs, multi-level pores) can also reduce coke precursor residence time. Finally, chemical poisoning stems from the irreversible adsorption of trace impurities (e.g., H_2_S_7_ Cl^−^) in the raw materials at the active site (e.g., 1 ppm H_2_S can form a Cu-S bond to block methanol adsorption) or damage to key interfacial structures (e.g., Cl^−^erosion of the Cu–ZnO interface) [29]. The role of the vector in this regard is mainly reflected in the following aspects: a strong and stable interface (e.g., Cu-ZnO) may provide some resistance; Carriers with zeolite effect (such as zeolite) can theoretically physically block macromolecular poisons from approaching the Cu site within the limit (but have limited effect on small molecules such as H_2_S). The strong adsorption of certain vectors to specific toxicants may also provide limited sacrificial protection. In particular, these three deactivation mechanisms are often coupled and synergistic under actual complex working conditions: the coarsening of particles caused by sintering exposes more low-reactive crystal planes, promotes CO adsorption and accelerates Boudouard carbon deposition. The formed carbon layer produces a local adiabatic effect, which further accelerates the sintering with the increase of temperature. Poison adsorption directly weakens the overall activity and regeneration ability of the catalyst [28]. Therefore, one of the core strategies for designing high-performance anti-deactivating copper-based catalysts is to deeply understand and use the differential effects of carrier materials on sintering resistance, surface acidity and alkalinity (regulating carbon deposition paths) and interfacial stability, so as to achieve synergistic optimization of thermodynamic stability and surface chemical properties.

Although sintering, carbon deposition, and chemical poisoning all contribute significantly to the deactivation of copper-based catalysts, sintering is often the dominant mechanism in most industrial applications, especially at high temperatures (>300 °C), as it results in a significant decrease in active site density. Carbon deposits and chemical poisoning are usually less affected and more dependent on reaction conditions and raw material purity. For example, chemical poisoning may become more pronounced at low temperatures or with sulphur-containing raw materials. In terms of quantitative comparison, experimental data shows a “conversion rate decay of 62%” mentioned above. However, in one experiment, the sintering of the unmodified Cu/Al_2_O_3_ catalyst after 100 h at 300 °C resulted in an increase in Cu particle size from 4.2 nm to 15.6 nm and a 62% reduction in methanol conversion, suggesting that it was the main deactivation mechanism. In contrast, the decay of activity due to carbon deposition is about 20%, while chemical poisoning (e.g., Cu-S bond formation due to 1 ppm H_2_S) accounts for only about 10% [27].

Considering that deactivation mechanisms vary with reaction conditions, significance analysis under different conditions is essential. At high temperatures (>300 °C), sintering is often the dominant mechanism for the deactivation of copper-based catalysts, as it results in significant coarsening of Cu particles and a decrease in active site density. With low temperatures (<250 °C) or with poisons (e.g. H_2_S, Cl^−^), the effects of carbon deposition and chemical poisoning may exceed those of sintering. For example, raw materials containing 1 ppm H_2_S can rapidly form Cu-S bonds, which directly block methanol adsorption, making chemical poisoning the primary deactivation factor [42]. It is worth noting that these three deactivation mechanisms are often coupled and synergistic with each other under actual working conditions. For example, particle coarsening caused by sintering will expose more low-reactive crystal planes, which will aggravate CO adsorption and carbon deposition. The thermal insulation layer formed by carbon deposition will heat up locally, further accelerating sintering. Chemical poisoning directly destroys the active site. Nonetheless, sintering is often considered to be the most significant mechanism of deactivation, as it is the most severe direct disruption of active site density, while the effects of carbon deposition and chemical poisoning are more dependent on specific reaction conditions and raw material properties.

### 2.3. Dynamic Coupling Effect of Reaction Conditions and Operating Parameters

The activity and stability of copper-based catalysts are not only affected by their intrinsic physicochemical properties, but also closely related to the dynamic coupling of reaction conditions and operating parameters, which has a significant regulatory effect on catalytic performance by changing the reaction path, surface adsorption behavior, and mass and heat transfer processes [43]. On the one hand, increasing the temperature can accelerate the methanol dehydrogenation kinetics and on the Cu/ZnO/Al_2_O_3_ catalyst (Cu particle size 4.2 nm, 250 °C), the H_2_ yield increased from 2.1 to 3.0 mol/(g_cat·min), but when the temperature exceeds the Tammann temperature of Cu^0^, the mobility of Cu^0^ particles increases sharply, resulting in an exponential increase in the sintering rate [44,45]. On the other hand, local hot spots induced by temperature gradients can exacerbate the support phase transition, weaken the metal–support interaction (SMSI), and lead to the stripping of active components [46]. Low-pressure conditions are conducive to the diffusion of gaseous reactants into the catalyst pores, but they will reduce the liquid phase coverage of H_2_O and inhibit the water–gas shift reaction (WGSR), CO selectivity increased from 0.8% to 2.5% on Cu/Al_2_O_3_ catalyst (Cu particle size 5.1 nm, 240 °C) [47]. Although high-pressure conditions can promote WGSR, they may induce the oxidation of Cu^+^ particles, especially when the molar ratio of H_2_O/CH_3_OH is >3 [48]. The formation of Cu_2_O can account for more than 40% of the surface copper species, significantly reducing the dehydrogenation activity.

Fluctuations in feedstock composition are a common challenge in industrial scenarios. Trace amounts of formic acid in methanol feedstock are acid-catalyzed dehydration to form dimethyl ether (DME), which has a strong adsorption to cover the active site, 20–30% reduction in initial conversion on Cu/ZnO catalyst (Cu particle size 6.3 nm, 220 °C) [49]. The dynamic change of the H_2_O/CH_3_OH ratio will restructure the surface adsorption layer, and the excessive coverage of hydroxyl groups (-OH) at high water content may block the methanol adsorption channel. However, the appropriate amount of H_2_O can inhibit the deep adsorption of CO through competitive adsorption, on the Cu/ZrO_2_ catalyst (Cu particle size 4.5 nm, 260 °C), the carbon deposition generation rate decreased from 0.12 to 0.06 g/(g_cat·h) [44,50]. The optimization of space velocity (GHSV) needs to balance activity and mass transfer limitations. The residence time of reactants is extended at low space velocity, and 98% methanol conversion on Cu/ZnO/Al_2_O_3_ catalyst (Cu particle size 5.0 nm, 230 °C) [51]. However, the long contact time will promote side reactions, resulting in the polymerization and coking of formaldehyde intermediates. Although high space velocity can inhibit side reactions, the actual conversion rate is reduced to less than 70% due to limited mass transfer.

In addition, abrupt changes in temperature and pressure caused by start–stop cycles can induce catalyst structural fatigue. For example, frequent hot and cold cycles can cause microcracks in Cu/ZnO/Al_2_O_3_ supports, disrupting the active site distribution and accelerating the loss of ZnO components [52]. It is important to note that these parameters do not act in isolation, but exacerbate performance degradation through nonlinear coupling. For instance, when the reaction temperature increases synchronously with the H_2_O/CH_3_OH ratio, the oxidation and sintering of Cu particles occur synergistically, and the deactivation rate can reach 1.8 times that of a single factor. Similarly, the combination of high pressure and high space velocity will aggravate the two-phase flow scouring in the pores, resulting in mechanical wear. In view of such complex coupling effects, dynamic operation strategies have been proposed in recent years [53,54]. For example, the standard deviation of the H_2_ yield of Cu/ZrO_2_ catalyst under fluctuating conditions can be reduced from 12% to 4% by adjusting the temperature and feed ratio in real time through model predictive control (MPC) [55]. Alternatively, the pulsed feed mode can be used to maintain surface cleanliness by taking advantage of transient adsorption differences, extending continuous operating life from 500 h to 1200 h [56]. However, the multi-parameter strong coupling modeling of industrial-grade reactors still faces challenges such as high-dimensional data scarcity and real-time control lag. Therefore, it is necessary to combine digital twin technology and in situ sensing network to achieve accurate optimization.

## 3. Design Strategies for Copper-Based Nanocatalysts

### 3.1. Nanostructure Design and Dispersion Control

**Figure 3:**
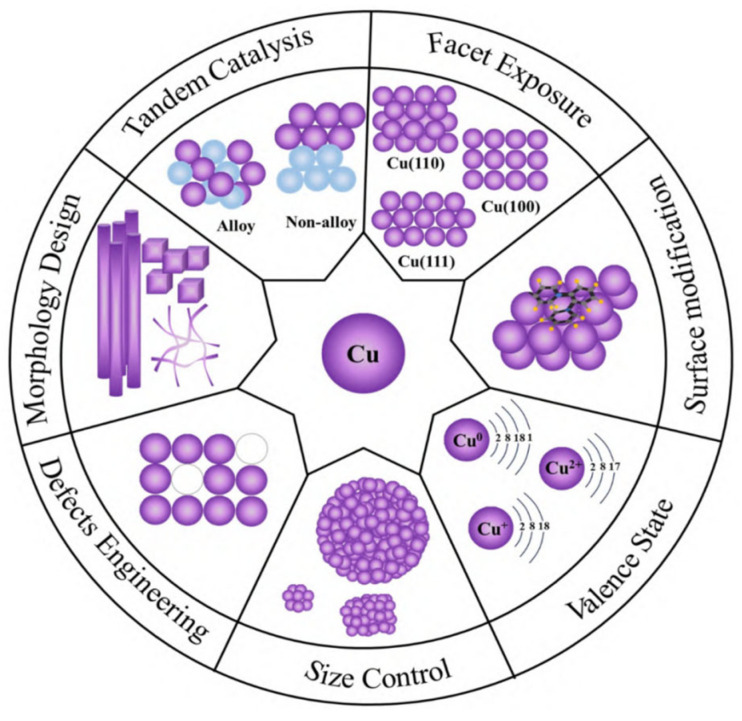
Schematic diagram of the catalyst design strategy.

The nanostructure design needs to be deeply coupled to the reaction mechanism. As shown in Figure 3, when Cu particle size < 5 nm, high-index crystal plane (e.g., Cu(110)) exposes stepped edge sites, and their low-coordination Cu atoms optimize methanol C-O bond cleavage pathways (energy barrier down to 0.8 eV). The mesoporous confinement effect (3–5 nm channel) can simultaneously inhibit sintering and regulate intermediate diffusion. In today’s cutting-edge research of catalytic science and technology, copper-based catalysts have become the focus of scientific researchers because of their potential advantages in many chemical reactions, such as methanol reforming and syngas conversion. However, how to further improve the activity and stability of copper-based catalysts to meet the growing demand for industrial production is still a key problem to be solved. In this context, nanostructure design and dispersion control, as the core strategies to optimize the performance of copper-based catalysts, are gradually highlighting their irreplaceable importance.

Through precise nanostructure design, the physicochemical properties of copper-based catalysts can be profoundly changed at the microscopic level, and their catalytic performance can be significantly improved. Among the many advanced synthesis methods, co-precipitation, sol-gel, and microemulsion methods stand out [57]. These methods, with their unique reaction mechanisms and condition control, enable the precise synthesis of highly dispersed Cu nanoparticles with tight particle sizes in the ideal range of less than 5 nm. Such a small particle size leads to significant changes in the surface atomic environment of Cu nanoparticles, and a large number of low-coordination atoms are exposed to the surface. These low-coordination atoms have unique electronic structures and chemical activities, which can greatly optimize the adsorption behavior of methanol on the surface of the catalyst, making it easier to react, and significantly enhance the catalyst’s ability to cleave C-O bonds, creating favorable conditions for the efficient conduct of catalytic reactions [58] (as shown in Figure 3).

Taking the mesoporous Cu/ZnO catalyst prepared using a metal-organic framework (MOF) template as an example, the unique porous structure and tunable composition of the MOF material provide an ideal template for the preparation of catalysts with specific nanostructures [59]. In the mesoporous Cu/ZnO catalysts prepared by this method, the uniformly distributed mesoporous structure not only provides a channel for rapid diffusion of reactants and products, but also greatly increases the specific surface area of the catalyst, thereby exposing more active sites [60]. Experimental studies have shown that the mesoporous Cu/ZnO catalyst exhibits excellent activity in the methanol conversion reaction, and the methanol conversion rate of the mesoporous Cu/ZnO catalyst (Cu particle size 3.8 nm, 240 °C) prepared by MOF template was 35% higher than that of the traditional catalyst compared with traditional catalysts [61]. This significant performance improvement is a testament to the potential and effectiveness of precise nanostructure design in optimizing catalyst activity.

In addition to the nanostructure design, dispersion control is also an indispensable key factor to improve the performance of copper-based catalysts. In the actual catalytic reaction, the agglomeration and sintering of catalyst particles often lead to the reduction of active sites and the degradation of catalyst performance. In order to solve this problem, the physical constraint strategy came into being. For example, a dense SiO_2_ shell is applied to the surface of Cu nanoparticles, or Cu particles are confined to zeolite with a special pore structure [50]. This physical restraint method can effectively limit the migration and aggregation of Cu particles under high-temperature reaction conditions, so as to greatly increase the sintering temperature threshold of Cu particles to more than 500 °C. After 300 h of continuous operation of the Cu catalyst in the SiO_2_ confined region (initial particle size 3.5 nm, 300 °C), the particle size increased by only 1.4 nm and the activity decay rate <10%. The standard deviation of Cu particle size < 0.5 nm and the carbon deposition rate were as low as 0.02 g/(g_cat·h) in the Cu–CeO_2_ core-shell structure during 500 h of continuous operation [62]. This excellent stability performance is due to the effective regulation of the dispersion of Cu particles by the physical confinement strategy, which enables the catalyst to maintain good structural stability and activity during the long-term reaction process, which provides a strong guarantee for the continuous and stable operation of industrial production.

### 3.2. Surface Nanoengineering and Interface Synergy

In the field of catalysis, although copper-based catalysts have shown many excellent properties, the problem of deactivation has always been a bottleneck that limits their wide application and long-term efficient operation. The emergence of surface and interface nanoengineering has brought a new dawn to break through this dilemma, and has become a key research direction to improve the performance of copper-based catalysts.

**Figure 4:**
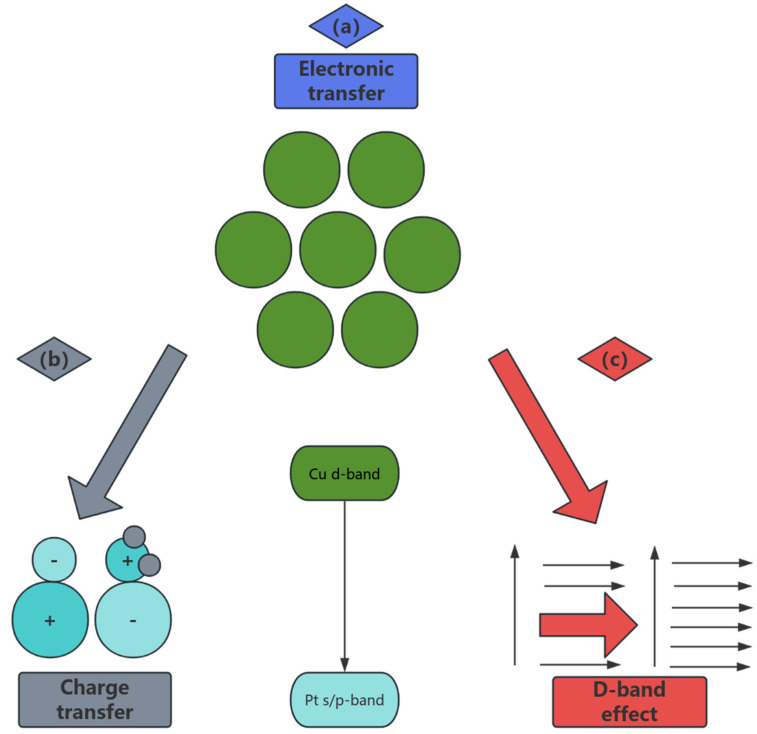
Schematic diagram of the electron effect in alloy catalysis (**a**) and the resulting charge transfer (**b**) and band change (**c**).

Atomic layer deposition (ALD), as an important means of surface nanoengineering, plays a unique role in optimizing the performance of copper-based catalysts [63]. This technology enables the precise preparation of ultra-thin Al_2_O_3_ coatings with a controlled thickness of about 2 nm. This ultra-thin coating works wonders as it acts as a strong and breathable “protective clothing” and is excellent at inhibiting the sintering of Cu particles [64]. We know that Cu particles are susceptible to sintering in high-temperature reaction environments, resulting in a reduction in active sites and degradation of catalyst performance. However, the Al_2_O_3_ coating prepared by ALD can effectively prevent the proximity and fusion of Cu particles, resulting in a significant reduction in the Al_2_O_3_ coating (thickness 2 nm) prepared by ALD reduced the sintering rate of Cu nanoparticles (initial particle size 4.0 nm) by 80% at 350 °C [65]. At the same time, this coating does not hinder the free diffusion of small molecules on the catalyst surface, ensuring that reactants and products can be smoothly adsorbed, reacted, and desorption on the catalyst surface, maintaining the efficient progress of the catalytic reaction [66] (as shown in Figure 4).

In addition to surface coating technology, the construction of heterogeneous interfaces is also the core content of interface synergy strategy. By carefully designing and constructing heterogeneous interfaces such as Cu–CeO_2_ or Cu-ZnO, the unique physicochemical properties at the interfaces can be used to optimize the performance of catalysts. Taking the Cu–CeO_2_ heterogeneous interface as an example, the oxygen vacancies present at the interface play a crucial role. These oxygen vacancies act as “acceleration engines” for chemical reactions, facilitating the rapid migration of hydroxyl groups on the surface of the catalyst [67]. In reactions involving CO oxidation, this rapidly migrating hydroxyl group can rapidly react with CO and oxidize it to CO_2_, and the selectivity of CO can be effectively controlled to <1%, which greatly improves the selectivity and efficiency of the reaction [68].

In addition, the Pt monoatomic layer-modified Cu surface shows us another wonderful interfacial synergy. When the Pt monoatomic layer is modified on the Cu surface, a bifunctional site is formed. These two functional sites work together to facilitate the dehydrogenation process and the other to facilitate the water–gas shift reaction, which synergistically improves the efficiency of the entire catalytic reaction [69]. This design concept of bifunctional sites provides a new way of thinking and methods for the development of high-performance copper-based catalysts.

Surface nanoengineering and interface synergistic strategies provide a variety of effective ways to solve the problem of deactivation of copper-based catalysts. Through the precise coating of ALD technology and the clever construction of heterogeneous interfaces and dual-functional sites, we are able to not only inhibit the sintering of catalysts, but also dynamically adjust the state of the active site to improve the selectivity and efficiency of the reaction [70]. With the continuous research of these technologies, it is expected to further improve the performance of copper-based catalysts and promote their wide application in more industrial fields.

### 3.3. Multivariate Collaborative Optimization of Nanocomposites

**Figure 5:**
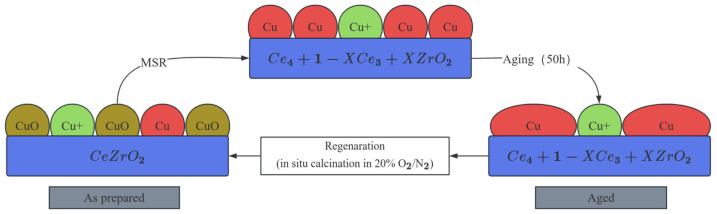
Schematic diagram of the surface state of CeO_2_/ZrO_2_ catalyst under different conditions.

Beyond the modulation of the d-band center, alloying fundamentally alters the reaction pathway by influencing the adsorption geometry and stability of key intermediates. For instance, in the Cu-Ni system, Ni atoms induce a partial positive charge on neighboring Cu atoms due to electron transfer. This electrophilic Cu^0^ + site stabilizes the oxygen lone pair of the methoxy intermediate (CH_3_O), strengthening the Cu-O bond by approximately 0.15 eV compared to pure Cu. This stabilization lowers the energy barrier for the subsequent C-H bond cleavage in CH_3_O, facilitating its dehydrogenation to formaldehyde (HCHO). Furthermore, the Ni-rich regions exhibit enhanced affinity for oxygen, promoting the dissociation of H_2_O to hydroxyl groups (OH) at significantly lower activation energies. These mobile OH species readily migrate to adjacent Cu sites where CO is adsorbed. The presence of Ni modifies the electronic structure, weakening the CO adsorption strength (as indicated by the d-band shift), and, crucially lowering the energy barrier for the reaction CO + OH → COOH (a key step in the water–gas shift pathway towards CO_2_ formation) by ~0.35 eV. This synergistic effect not only accelerates the desired WGSR but also diverts CO away from the Boudouard reaction pathway (2CO → C + CO_2_), effectively suppressing carbon deposition at its source. In the Cu-Ni system, the Ni-rich region reduces the H_2_O dissociation energy barrier, while the Cu-rich region weakens the CO adsorption energy, and the Boudouard carbon deposition reaction is synergistically inhibited. The multivariate synergistic optimization of nanocomposites has achieved an overall improvement in activity, selectivity and stability by precisely controlling the composition, interface and microstructure of copper-based catalysts. In the alloying strategy, the electronic synergy effect of bimetallic nanoparticles has become the core breakthrough point. For example, the Cu-Ni alloy system significantly adjusts the electronic structure of Cu’s d-band through the introduction of Ni: theoretical calculations show that the center of Ni’s d-band is shifted down by 0.4 eV compared with pure Cu, resulting in a decrease in CO adsorption energy from −1.2 eV to −0.8 eV, effectively inhibiting the Boudouard reaction (2CO → C + CO_2_), and reducing the carbon deposition rate from 0.15 g/(g_cat·h) to 0.07 g/(g_cat·h) [71]. At the same time, the Cu-rich region on the surface of Cu-Ni nanoparticles preferentially adsorbs methanol molecules, while the Ni-rich region accelerates the dissociation of H_2_O to form hydroxyl radicals through strong oxygen affinity (· OH), which promoted the kinetics of the water–gas shift reaction (WGSR), increased the H_2_ yield to 3.5 mol/(g_cat·min), and stabilized the CO selectivity below 0.5% [72] (as shown in Figure 5). In addition, the Cu-Co nanocomposite catalyst dynamically adjusts the interfacial oxygen vacancy concentration through the redox properties of Co, reducing the WGSR activation energy from 65 kJ/mol to 45 kJ/mol at 250 °C, with a CO_2_ selectivity of up to 99.1% [71]. This “bimetallic active site partitioning” design not only optimizes the reaction pathway, but also inhibits the migration of Cu particles through the pinning effect of Co, and the particle size only increases from 3.2 nm to 4.1 nm after 300 h of operation, and the activity decay rate is reduced <8%.

**Figure 6:**
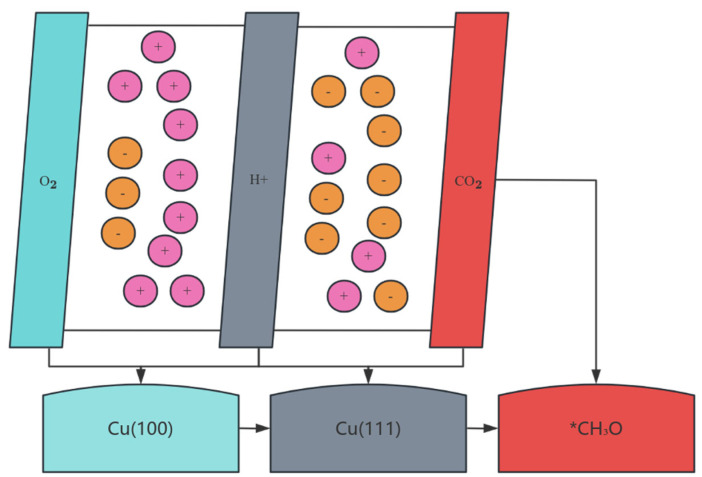
The principle of electrochemical CO_2_RR.

The nanoscale doping of the cocatalyst further strengthened the anti-deactivation ability of the copper-based system. The introduction of ZnO nanoparticles stabilizes Cu^+^ species and regulates Cu^0^/Cu^+^ dynamic equilibrium by forming a Cu–ZnO interface strong interaction (SMSI) [73]. In-situ XAFS studies demonstrate that the electron donor effect of ZnO increases the Cu^+^ ratio from 30% to 45%, promotes the stabilization of methoxy intermediates, and reduces the methanol dehydrogenation barrier by 0.3 eV, achieving a conversion rate of 95% at 220 °C.The doping of CeO_2_ nanocrystals dynamically regulates the oxygen vacancy concentration through the Ce^3+^/Ce^4+^ redox cycle, accelerating the CO oxidation pathway [74]. For example, for every 1 × 10^18^ cm^−3^ oxygen vacancy density increase at the Cu–CeO_2_ interface, the CO oxidation rate increases by 12%, and the CO selectivity decreases to <0.3%. In addition, the acidic site of the Al_2_O_3_ nanocoating combined with the electron conduction properties of TiO_2_ nanoparticles to construct an “acid-base-electron bifunctional carrier”, which increased the Cu dispersion from 50% to 75%, inhibited the formation of dimethyl ether (DME) by-products, and reduced the carbon deposition rate by 60% [23] (as shown in Figure 6).

The multi-level pore carrier design significantly improves the reaction efficiency through nanoscale mass transfer optimization. This optimized mass transfer efficiency is due to the precise regulation of reactant diffusion paths and intermediate residence times by the multi-level pore structure [75]. For example, the three-dimensional interpenetrating channel network formed by MOF-derived hierarchical pore carbon (mesoporous 3–5 nm, macroporous 50–100 nm) greatly reduces the resistance of methanol molecule diffusion to the active site, and increases the contact frequency between reactants and the active site per unit time, thereby increasing the methanol diffusion flux to 5.2 × 10^−6^ mol/(m^2^·s) [28]. At the same time, the directional macroporous structure (e.g., from wood template) simulated the mass transfer network of plant vascular bundles, which significantly shortened the residence time of reaction intermediates (e.g., CO) in the micropores (reduced to 0.8 ms), effectively inhibited the side reactions that may lead to carbon deposition (e.g., Boudouard reaction), and further reduced the carbon deposition rate to 0.02 g/(g_cat·h) [29]. Therefore, the multi-stage pore design not only improves the intrinsic reaction rate, but also synergistically enhances the overall efficiency and long-term stability of the catalyst by inhibiting the deactivation pathways associated with mass transfer limitation [76]. For example, after 500 h of continuous operation at 300 °C, the Cu particle size of the ZIF-8-derived nitrogen-doped carbon-supported Cu catalyst increased from 3.5 nm to 4.3 nm, and the carbon deposition was as low as 0.03 g/(g_cat·h). The graphene/carbon nanotube composite supports use their high conductivity and hydrophobicity to accelerate electron transfer and reduce surface hydroxyl coverage, increasing the H_2_ yield to 4.1 mol/(g_cat·min) [77]. The latest studies have also shown that biomimetic hierarchical pore structures, such as oriented macropores derived from wood templates, can mimic the mass transfer network of plant vascular bundles, shorten the residence time of CO molecules in the micropores to 0.8 ms, and further reduce the carbon deposition rate to 0.02 g/(g_cat·h) [78].

The theoretical exploration of the synergistic mechanism provides a new perspective for material design. Density functional theory (DFT) calculations have revealed that the charge transfer at the Cu–ZnO interface forms an electron-deficient Cuδ^+^ site, which reduces the O-H bond dissociation barrier of H_2_O from 1.4 eV to 0.9 eV, significantly accelerating WGSR dynamics [79]. Molecular dynamics (MD) simulations further show that the mesoporous SiO_2_-confined Cu nanoparticles move up by 0.2 eV, increase the methanol dehydrogenation rate by 30%, and increase the CO diffusion energy barrier to 0.8 eV due to the electron enrichment effect, achieving a synergistic optimization of activity and stability [80]. By analyzing 2000 sets of experimental data, the machine learning model screened out the Cu-ZnO-CeO_2_-MgO quaternary system, whose ZnO/MgO molar ratio (2:1) suppressed the carbon deposition rate to 0.03 g/(g_cat·h) through acid-base synergy, and the methanol conversion rate was stable at more than 95% for a long time [81] (as shown in Table 2). These cross-scale theoretical-experimental linkage strategies are promoting the innovation of copper-based nanocomposites to the whole chain of “precise design, efficient preparation, and intelligent control”.

### 3.4. Catalytic Design of Electrochemical Reduction Systems

The electrochemical CO_2_ reduction reaction (CO_2_RR) uses a copper-based catalyst to convert CO_2_ into high value-added hydrocarbons, and its reaction path is fundamentally different from that of thermocatalytic MSR. As shown in Figure 6, CO_2_RR undergoes a multi-step proton-electron transfer process: CO_2_ is first adsorbed on the Cu surface to form COOH intermediates, and then dissociated to CO. Thereafter, *CO can be hydrogenated to produce C_2_ products (e.g., C_2_H_4_, ethanol) via the C-C coupling pathway, or to CH_4_ by hydrogenation. This pathway is strongly dependent on the electric double-layer structure: the electrolyte pH (e.g., alkaline conditions promote C-C coupling), the local CO_2_ concentration (regulated by the thickness of the diffusion layer), and the interfacial electric field strength (which affects *CO coverage) determine the product distribution (as shown in Figure 6). It is worth noting that the orientation of the Cu crystal plane is the decisive factor for selectivity: the Cu(100) crystal plane promotes *CO dimerization due to tetragonal symmetry, resulting in a C_2_H_4_ Faraday efficiency (FE) of 60–80% (−0.9 V vs. RHE, 1 M KOH). However, the Cu(111) crystal plane is more conducive to the formation of *CH_3_O, resulting in a >45% selectivity of CH_4_ [82]. However, competitive hydrogen evolution reactions (HER) are always a central challenge—the HER current density on the Cu surface can be up to three times that of CO_2_RR (about 120 mA/cm^2^) at an overpotential of −1.0 V, due to the fact that the H^+^ reduction barrier (0.2 eV) is lower than the *CO plus hydrogen barrier (0.8 eV) (as shown in Figure 6). In situ surface-enhanced Raman spectroscopy (SERS) confirmed that the Cu^+^/Cu^0^ dynamic equilibrium is critical for HER inhibition: the Cu_2_O-derived catalyst maintains a Cu^+^ ratio of 40–60% in the reaction, increasing ethanol selectivity to 45% by stabilizing the *OCH_2_ intermediate [83].

In view of the above challenges, the rational design of copper-based electrocatalysts focuses on three dimensions:

(1) Nanostructure engineering: constructing high-curvature surfaces to increase low-coordination active sites. For example, the dendrite Cu nanowire (20 nm diameter) increased the FE of C_2_ product to 75% (−1.1 V, 0.1 M KHCO_3_) due to its abundant edge sites, which was 2.3 times higher than that of planar electrodes. Oxygen-vacancy-mediated confinement effects have also been shown to reduce the C-C coupling energy barrier: for every 1 × 10^21^ cm^−3^ increase in oxygen vacancy concentration in the CeO_2_-x/Cu heterostructure, the rate of C_2_H_4_ formation is increased by 28 percent (from 0.75 eV to 0.52 eV) [84].
(2) Electron control of the carrier: The nitrogen-doped carbon support enhances the Cu electron density through p-d orbital hybridization, weakens the adsorption strength of *CO (the adsorption energy increases from −1.5 eV to −0.9 eV), thereby promoting the desorption and dimerization of *CO. Experiments have shown that the C_2_H_4_/ethanol ratio can be optimized from 1:1 to 4:1 at a pyridine nitrogen content of >5 at% [83].
(3) Electrolysis System Synergy: Membrane Electrode Assembly (MEA) Design Breaks Through Mass Transfer Limitations. In a solid-state electrolyte reactor, the gas diffusion layer of the Cu/PTFE composite cathode (50 μm thick) increases the CO_2_ flux to 200 mL/min·cm^2^ and the C_2_H_4_ yield to 1.2 A/cm^2^, while avoiding catalyst deactivation due to carbonate precipitation [24]. However, the industrial-grade conversion still faces a bottleneck: at a current density of >300 mA/cm^2^, the Cu catalyst has a lifetime of <100 h due to cathodic corrosion. The pulsed potential strategy (−0.8 V/−0.2 V alternation) extends the stability to 500 h by periodically reducing copper oxide, but with a 15% loss of energy efficiency [85]. In the future, it is necessary to combine machine learning to optimize the potential waveform and develop corrosion-resistant carriers (TiN-coated carbon paper) to realize the leap of CO_2_RR from the laboratory to the green hydrogen industry chain.

### 3.5. Green Nanosynthesis and Intelligent Regeneration Technology

Green nanosynthesis and intelligent regeneration technology provides a new paradigm for the sustainable development and recycling of copper-based nanocatalysts through innovative preparation processes and dynamic response mechanisms. In the field of green synthesis, biomass-derived carriers have become a research hotspot due to their advantages of low carbon, low cost and high performance. For example, rice husk ash is converted into a mesoporous SiO_2_ carrier by alkali-solubilization-gel method, and its abundant silicon hydroxyl group on the surface can form a uniform load with the Cu^2+^ precursor through electrostatic adsorption, and the dispersion of Cu nanoparticles reaches 88%, which is 40% higher than that of traditional commercial SiO_2_ supports, and the synthesis energy consumption is reduced by 70% [86]. The lignin-based porous carbon carriers were combined with high-temperature carbonization and KOH activation to construct a hierarchical pore structure, in which the surface oxygen-containing functional groups (-COOH and -OH) could anchor Cu nanoparticles (particle size 3.5 ± 0.3 nm), the lignin porous carbon carrier-supported Cu catalyst (Cu particle size 3.5 ± 0.3 nm) achieved a methanol conversion of 92% at 240 °C, and the carbon deposition rate was as low as 0.04 g/(g_cat·h) [87]. For example, using TiO_2_ nanowires as photocatalysts, Cu^2+^ precursors are efficiently reduced to Cu^0^ nanoparticles at 80 °C under ultraviolet light irradiation, with narrow particle size distribution and no need to add toxic reducing agents, reducing carbon emissions by 65%. By doping Ag nanoparticles as electron transport bridges, the visible light-driven Cu/Ag-TiO_2_ system achieves rapid reduction of Cu^2+^ under simulated sunlight, increases the particle dispersion to 92%, and exhibits excellent photothermal synergistic catalytic performance, the visible light-driven Cu/Ag-TiO_2_ system (Cu particle size 3.2 nm, 200 °C) achieved an H_2_ yield of 4.3 mol/(g_cat·min) [83] (as shown in Table 3).

**Figure 7:**
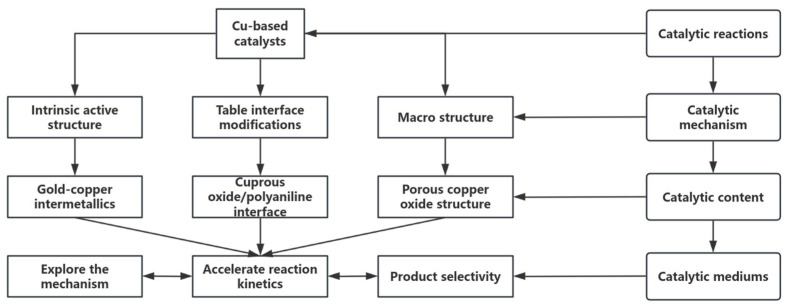
The schematic diagram of the research contents.

The intelligent regeneration technology significantly extends the life of the nanocatalyst through dynamic response materials and precise control strategies. Temperature-sensitive polymer coatings can be adapted to adjust the reaction conditions through phase change behavior: at the reaction temperature, PNIPAM shrinks to expose hydrophobic pores, which promotes the diffusion of methanol molecules to the Cu active site, increasing the diffusion rate by 2.3 times, and, when it is shut down for cooling, it expands to cover the orifice, isolates H_2_O and CO_2_ in the air, prevents the oxidation of Cu nanoparticles, and improves the stability of the start–stop cycle by a factor of 3 [88]. The Cu catalyst supported by light-responsive MOF materials (such as UiO-66-NH_2_) produces a local thermal effect (ΔT ≈ 50 °C) under near-infrared irradiation, triggers the dynamic recombination of ligands, repairs the lattice defects caused by sintering in real time, and keeps the Cu particle size at 4.2 ± 0.4 nm after 500 cold and hot cycles, with an activity attenuation rate <5%. For carbon deposition deactivation, the biomimetic self-healing coating catalyzes the oxidation of carbon deposition to CO_2_ in the presence of H_2_O_2_ by embedding oxidase mimics, with a regeneration efficiency of 98% and no need to disassemble the reactor, with a single regeneration cycle of only 2 h [90] (as shown in Figure 7).

**Figure 8:**
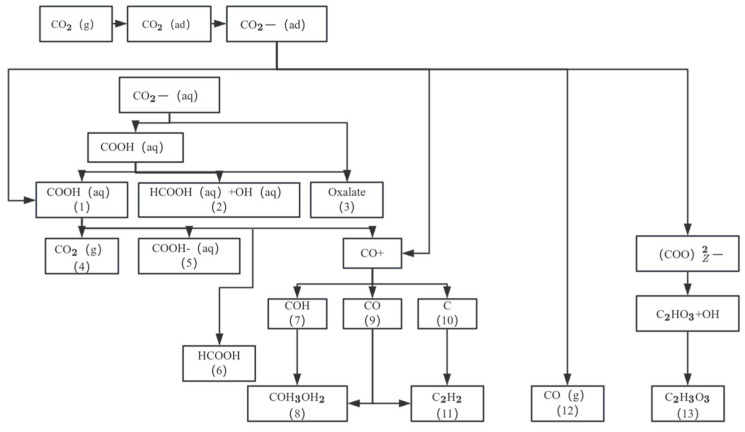
Selectivity of different metal electrodes for electrocatalytic CO_2_ reduction products.

In terms of regeneration process, the weak acid selective leaching technology can dissolve 90% of the Cu components in the deactivated catalyst by optimizing the leaching conditions, while retaining the ZnO/Al_2_O_3_ carrier structure, and then reloading the Cu nanoparticles by co-precipitation-microwave-assisted reduction method, and the activity recovery rate of the regenerated catalyst reaches 92%, which is significantly higher than that of the traditional high-temperature calcination method. In plasma-assisted regeneration, high-energy electrons are generated by glow discharge of Ar/H_2_ mixed gas, and the surface carbon layer is stripped and oxidized Cu species are reduced at room temperature, and the carbon deposition can be reduced from 8 wt% to 0.5 wt%, and the Cu^0^ ratio can be restored from 40% to 78% in 10 min [89]. In addition, the electrochemical regeneration method takes advantage of the redox reversibility of Cu^2+^/Cu^0^ to directly electrodeposit the CuO in the deactivated catalyst into highly dispersed Cu nanoparticles at a voltage of 0.5 V, reducing the regeneration cost by 50%. The Life Cycle Assessment (LCA) shows that the combined application of green nanosynthesis and intelligent regeneration technology can reduce the carbon emissions of copper-based catalysts by 60% and increase the utilization rate of raw materials to 85%. For example, the combination of rice husk ash carrier and photocatalytic reduction process consumes only 1.3 kWh per kilogram of catalyst preparation and reduces wastewater discharge by 90% [91]. In the future, the customized microbial directional synthesis of Cu nanoparticles (particle size 2–5 nm) through synthetic biology technology, or the directional assembly of multi-level pore structure carriers by 3D printing, will further promote the large-scale application of green nanocatalysts and provide key technical support for the low-carbon transformation of the hydrogen economy (as shown in Figure 8).

## 4. Advanced Characterization and Theoretical Calculation

### 4.1. In Situ Characterization Techniques

The rapid development of in situ characterization technology provides an atomic-level perspective for revealing the dynamic behavior of copper-based catalysts in methanol reforming reactions, enabling researchers to track the structural evolution of active sites and the dynamic adsorption process of reaction intermediates in real time [22]. In situ X-ray absorption fine structure spectroscopy (XAFS) can accurately analyze the valence state (Cu^0^/Cu ratio) and local coordination environment changes of Cu by monitoring the K absorption edge displacement and spread edge oscillation of Cu [21,92]. For example, some scholars have found that Cu^0^ is the dominant Cu species in Cu/ZrO_2_ catalysts, and the proportion of Cu gradually increases to 48% with the continuous introduction of H_2_O [93], confirming the key role of Cu in water–gas shift reactions. Further combined with in situ diffuse reflectance infrared Fourier transform spectroscopy (DRIFTS), the evolution of surface adsorption species can be captured. At 200 °C, methanol molecules are first adsorbed as methoxy groups (CH_3_O) by O-H bond cleavage, then C-H bonds are dissociated to form formyl groups (HCO), and finally HCO combines with surface hydroxyl groups (OH) to form CO_2_ and H_2_ [27,94]. This path is particularly significant in the Cu/ZnO/Al_2_O_3_ catalyst, and the characteristic peak strength of the formyl group is three times stronger than that of the pure Cu system, indicating that ZnO promotes the stabilization of intermediates [95].

In addition, the dynamic sintering process of Cu particles was directly observed by in situ transmission electron microscopy (TEM): under the reaction condition of 300 °C, the Cu particles in the unmodified Cu/Al_2_O_3_ catalyst were coarsened at a rate of 0.15 nm/min, while the coarsening rate of the confined Cu_2_SiO_2_ catalyst was reduced to 0.02 nm/min due to the physical barrier of the SiO_2_ shell [96,97,98]. When the ratio of H_2_O/CH_3_OH in the reaction gas increases from 1:1 to 3:1, the Cu_2_p3/2 binding energy shifts from 932.5 eV (Cu^0^) to 933.8 eV (Cu) [99], and the ratio of lattice oxygen (529.5 eV) to hydroxyl oxygen (531.2 eV) in the O1s spectrum changes from 1:0.7 to 1:1.3, which confirms the regulatory effect of H_2_O dissociation on the valence state of Cu [100]. In recent years, the coupling of Operando synchrotron radiation technology has further achieved the real-time correlation of “structure-performance”: for example, through the coupling of Operando XAFS with mass spectrometry (MS), it is found that the oxygen vacancy density in the Cu/CeO_2_ catalyst is linearly positively correlated with the CO_2_ generation rate, which provides a quantitative design basis for defect engineering [101]. However, the existing technology still faces the challenge of insufficient spatiotemporal resolution, for example, capturing femtosecond-level ultrafast reaction processes relies on cutting-edge facilities such as free electron laser (XFEL), and its application is still in the exploratory stage [102].

### 4.2. DFT Calculations

Density functional theory (DFT) calculations provide a theoretical foundation for rational design by simulating the electronic structure, surface reaction path, and energy changes of copper-based catalysts [103]. In terms of active site identification, researchers found that the adsorption energy of low-coordination Cu atoms (such as step edge or corner sites) to methanol (−0.85 eV) was significantly higher than that of flat surfaces (−0.52 eV) [104], and the C-O bond cleavage energy barrier decreased from 1.2 eV to 0.8 eV [105], explaining the essential reason for the high activity of small-sized Cu particles. In the study of alloying effects, the calculation of the d-band center on the Cu-Ni(111) surface shows that the introduction of Ni moves the d-band center from −2.3 eV (pure Cu) to −2.7 eV [106], weakening the CO adsorption energy and inhibiting the formation of carbon deposition, which is consistent with the experimental observation of a 50% reduction in carbon deposition [107]. The simulation of carrier interaction showed that the charge at the Cu/ZrO_2_ interface shifted by 0.15 e^−^ from Cu to ZrO_2_, forming an electron-deficient CuO site that promotes the dissociation of the O-H bond of H_2_O (the energy barrier decreased from 1.4 eV to 0.9 eV) [82]. However, due to the existence of oxygen vacancies at the Cu/CeO_2_ interface, the activation energy of the CO oxidation path was reduced to 0.6 eV, which is in good agreement with the measured CO selectivity of <1% [84].

In addition, the microkinetic model successfully predicted the methanol conversion deviation of the Cu/ZnO/Al_2_O_3_ catalyst in the range of 220–300 °C by integrating DFT energy data with the macroscopic rate equation, with a deviation of less than 5% [24]. For example, Zhao et al. (2025) quantified the contribution ratio of Cu^0^ to the total reaction rate by constructing a kinetic network of 12 motive reactions, and guided the synthesis of a gradient Cu^+^ distribution catalyst to increase the H_2_ yield by 22% [108]. However, DFT calculations still face two major limitations: one is that model simplification may lead to prediction bias, and the other is that the computational cost limits the simulation of large systems or long-term processes. For instance, the Cu–ZnO interface model, based on neural networks, can improve computational efficiency by 10 times while maintaining an energy error of less than 0.05 eV, opening up a new avenue for multi-scale catalyst design [85].

### 4.3. Synergistic Optimization Mechanism of Activity and Stability

In methanol reforming for hydrogen production, the activity and stability of copper-based catalysts often present complex competition or synergistic relationships, and advanced characterization and computational methods provide key tools to reveal their intrinsic relationships [109]. For example, quantum mechanical (QM) calculations show that the methanol dehydrogenation barrier at the Cu(111) surface step site is significantly lower than that on the flat surface, but the exposed low-coordination Cu atoms are also more likely to adsorb CO intermediates, which in turn triggers carbon deposition through the Boudouard reaction [110]. Molecular dynamics (MD) simulations show that, when Cu nanoparticles are encapsulated in mesoporous SiO_2_, the CO diffusion energy barrier increases from 0.4 eV to 0.8 eV, and while carbon deposition is suppressed, the electron enrichment caused by the confinement effect shifts the center of Cu’s d band up by 0.2 eV, and the methanol dehydrogenation rate increases by 30% [111,112]. In situ XAFS experiments further verified that the Cu@SiO_2_ catalyst maintained a stable Cu^0^/Cu^+^ ratio of 6:4 after running at 300 °C for 200 h, while the Cu^0^ ratio in the traditional Cu/Al_2_O_3_ catalyst was reduced from 70% to 40%, and the activity decay rate was improved from 58% to 12% [113].

For example, the ML model based on Bayesian optimization screened the Cu-ZnO-CeO_2_-MgO quaternary system from 2000 sets of experimental data, and its ZnO/MgO molar ratio (2:1) was found to be effective in maintaining methanol conversion through acid-base synergy under the premise of 95% (250 °C) [114]. This resulted in a reduction of the carbon deposition rate from 0.12g/(g_cat·h) to 0.03 g/(g_cat·h) [115]. Reinforcement learning (RL)-driven optimization of synthesis parameters also found that pH gradient control (initial pH = 10.5, final pH = 8.0) during co-precipitation could simultaneously increase the Cu dispersion (from 45% to 68%) and the crystallinity of the ZnO-Al_2_O_3_ support, and increase the anti-sintering temperature threshold of the catalyst by 80 °C [116]. However, the synergistic improvement of activity and stability still faces challenges. DFT calculations show that although oxygen vacancies at the Cu–CeO_2_ interface can promote H_2_O dissociation (activation energy is reduced to 0.5 eV), excessive vacancies (>1.2 × 10^19^ cm^−3^) will lead to electron depletion of Cu particles and weaken the methanol adsorption capacity [117]. For instance, periodic local heating (50 °C pulse) of the photothermal effect can eliminate carbon deposition in situ, while maintaining the Cu^+^/Cu^0^ dynamic equilibrium under the action of photogenerated carriers, achieving an activity recovery rate of >90% [118]. In the future, by constructing an “activity-stability descriptor” database, combined with high-throughput computation and automated experimental verification, it is expected to establish rational design criteria for copper-based catalysts and overcome the limitations of traditional trial-and-error methods [119].

The above-mentioned theoretical simulations, in situ characterization and machine learning models jointly reveal the intrinsic mechanism and potential design principles of co-optimization of activity and stability of copper-based catalysts. However, translating these advanced ideas into real-world high-performance catalysts and validating their long-term performance is a critical step towards industrial applications. Although machine learning (ML) has shown great potential to accelerate catalyst screening and optimize synthesis parameters, its current application still faces significant challenges:

(1) Scarcity and bias of high-quality training data: ML models rely heavily on large, high-quality, and standardized experimental data. However, data in the field of catalysis, especially those involving complex deactivation behaviors or industrial conditions, are often scattered, inconsistent, and limited in scale. There is a ‘gap’ between the data on ideal conditions at the laboratory scale and the complex data on mass and heat transfer, feedstock fluctuations, and long-term stability in industrial-scale reactors, resulting in increased bias in model predictions when scale-up or real-world applications.
(2) Limited model transferability: Models trained on specific catalyst systems or reaction conditions are often difficult to directly generalize to new systems with different chemical spaces or operating conditions. Industrial MSR involves complex dynamic processes such as raw material fluctuations, start–stop cycles, and impurity influences, and the current ML models have limited generalization capabilities for such ‘out-of-distribution’ scenarios.
(3) Lack of predictive interpretability: Complex ‘black box’ models provide highly accurate predictions, but they struggle to provide physicochemical insights into ‘why’ specific combinations or parameters are better. This hinders mechanistic understanding and rational catalyst design based on ML results.
(4) Computational cost and real-time control delay: The full-scale simulation of industrial reactors with strong multi-parameter coupling is extremely expensive. Although ML-based real-time optimization control can improve dynamic stability, it still faces the problem of data processing and decision-making delay under the high-dimensional space and rapid response requirements.

Table 4 summarizes the comprehensive performance metrics of representative copper-based catalysts with different design strategies in methanol vapor reforming reactions, including preparation methods, reaction conditions, hydrogen yield, carbon monoxide selectivity, and critically long-term stability (lifetime). These data visually demonstrate the significant breakthroughs in the current optimal catalyst system to achieve high activity and ultra-long lifetime, and provide a key reference for evaluating the practical effects of different design strategies and guiding the development of industrial-grade catalysts.

### 4.4. Dynamic In Situ Multimodal Characterization and Cross-Scale Theoretical Modeling

The improvement of the activity and stability of copper-based catalysts in methanol reforming reactions essentially depends on the accurate analysis of their dynamic surface chemical behavior and bulk phase structure evolution, and the combination of dynamic in situ multimodal characterization technology and cross-scale theoretical modeling is promoting the paradigm shift from static description to real-time regulation in this field [120]. Dynamic in situ multimodal characterization reveals the dynamic response mechanism of catalysts at atomic-molecular-mesoscopic scales through the hyphenated complementarity technique. For example, synchrotron radiation X-ray absorption spectroscopy (XAS) coupled with Raman spectroscopy can simultaneously track the evolution of the valence state of Cu and the adsorption kinetics of intermediate species on the surface. In Cu/ZrO_2_ catalysts, the Cu^0^ ratio increases rapidly from 65% to 78% at the beginning of the reaction, and Raman spectroscopy detects the instantaneous accumulation of formate species, indicating that the Cu^0^-dominated dehydrogenation pathway is dominant [121]. With the extension of the reaction time, the Cu^+^ ratio rises to 45%, and the surface hydroxyl group coverage increases, and WGSR gradually becomes the rate-controlling step [122]. Environmental transmission electron microscopy (ETEM) coupled with electron energy loss spectroscopy (EELS) further captured the nanoscale dynamic process [123]. In a 1 bar H_2_O/CH_3_OH atmosphere at 300 °C, the periodic migration of Zn atoms at the Cu/ZnO interface dynamically stabilized Cu^+^ species and promoted CO_2_ desorption through the formation of transient Zn-O-Cu bridging structures, which was directly related to the increase in WGSR activity [124].

Cross-scale theoretical modeling integrates quantum mechanics (QM), dynamics Monte Carlo (kMC), and computational fluid dynamics (CFD) to construct a complete prediction chain from electronic structure to reactor performance. For example, QM calculations show that the methanol dehydrogenation energy barrier at the Cu(110) surface step site is 31% lower than that at the planar site, while kMC simulations show that the H_2_ yield can be increased to 3.5 mol/when the density of such highly active sites is increased to 5 sites/nm^2^ [125]. Further coupled with the CFD model, it is found that the non-uniformity of the active site distribution in the industrial-grade tubular reactor will lead to a 40% difference in local mass transfer efficiency, and the flow field distribution needs to be optimized by gradient pore design. Machine learning-enhanced multimodal data fusion provides a new tool for cross-scale association. For example, the graph neural network-based model encodes XAS valence data, ETEM structure images, and reaction kinetic parameters into unified eigenvectors. This successfully predicts the dynamic deactivation trajectory of catalysts and guides the synthesis of Cu–CeO_2_ core-shell structures with self-healing functions [126]. These structures synergize with CeO_2_ oxygen vacancies through the confinement effect of ZIF-8 shells. At 500, the standard deviation of Cu particle size was maintained at <0.5 nm and the carbon deposition rate was as low as 0.02 g in continuous operation [127].

However, there are still three major challenges in this field: first, the signal-to-noise ratio (SNR) for in situ characterization under extreme conditions is insufficient. For example, at a pressure of 10 bar, the X-ray scattering background intensity increases by a factor of 3, resulting in a relative error of Cu particle size analysis increasing from 5% to 15% [113]. Second, the computational cost of cross-scale models is high, and full-scale simulation of industrial reactors consumes hundreds of millions of CPU hours, far exceeding the existing computing power. Third, there is a lack of standardization and sharing mechanisms for multimodal data, and differences in characterization protocols in different laboratories lead to data heterogeneity, which hinders the construction of global models [128]. To this end, researchers are developing a distributed computing framework based on federated learning, which realizes cross-institutional collaborative modeling under the premise of protecting data privacy through encrypted data sharing and local model aggregation. At the same time, the combination of quantum computing and variational autoencoder (VAE) is expected to improve the efficiency of cross-scale simulation by 100 times, laying the foundation for the realization of real-time digital twin catalytic systems.

## 5. Challenges and Prospects

### 5.1. Problems That Still Exist in Current Research

Copper-based catalysts have made significant progress in the field of methanol reforming for hydrogen production. However, their industrial application still faces multiple bottlenecks. Firstly, the contradiction between structural consistency and cost control in large-scale preparation is prominent: for example, although the confined core-shell structure can effectively inhibit sintering, the template synthesis step is cumbersome, and the material cost increases by 30–50% due to noble metal doping [27,95]. However, low-cost processes such as co-precipitation are difficult to accurately control the size distribution of Cu particles, resulting in a difference in activity of more than 15% between batches [129]. Secondly, the long-term stability under complex working conditions is insufficient: most of the existing studies are based on ideal conditions in the laboratory, and impurities such as sulfur and chlorine contained in actual raw materials will irreversibly adsorb to the active site [130]. In addition, temperature fluctuations in the reactor will induce dynamic reconstitution and support phase transformation of Cu particles, accelerating interfacial desorption. Thirdly, the development of catalyst regeneration technology is lagging behind: the traditional regeneration method of carbon deposition deactivation catalyst is easy to lead to Cu oxidation and carrier sintering, and the activity after regeneration is only 60–70% [131]. However, chemical cleaning may damage the interface structure of Cu-ZnO and cause secondary deactivation. Fourth, the deviation between the theoretical model and the actual system remains unresolved: DFT calculations are usually based on an ideal surface model, ignoring support defects, surface hydroxyl group coverage, and reaction fluid dynamics [30]. Although machine learning models can speed up screening, their training data are mostly from laboratory tests, which is difficult to cover the coupling effect of mass and heat transfer in industrial-grade reactors.

### 5.2. Future Directions

In order to break through the above bottlenecks, research and development of copper-based catalysts needs to evolve in the direction of multidisciplinary and dynamic intelligent control. First, multi-scale collaborative design: combined with precise preparation technologies such as atomic layer deposition and microfluidic synthesis, the cross-scale matching of active sites, carrier pores, and reactor flow fields can be realized. Second, dynamic active site control: the surface state of the catalyst is adjusted in real-time by using light, electricity, and magnetic external fields. As shown by near-field infrared thermal imaging, 980 nm laser irradiation can instantaneously increase the local temperature of Cu/CeO_2_ by 50 °C, trigger the Ce^3+^/Ce^4+^ redox cycle, dynamically remove carbon deposition, maintain Cu^+^/Cu^0^ equilibrium, and extend the single regeneration cycle to 500 h [31,132]. Third, a machine learning-driven closed-loop development system: build a full-factor database covering synthetic parameters, structural features, and performance data, and mine implicit structure-activity relationships through deep learning [42]. The European Union’s “Catalytic Earth” project (2023) has realized cloud-based AI-assisted design, shortened the development cycle of new materials to three months, and predicted the activity error of <8% [25]. Fourth, develop self-healing carriers that release interfacial stress via lattice deformation during thermal cycling, or design responsive coatings for autonomous carbon removal when deposition reaches a threshold [26]. In addition, it is necessary to cover the entire chain of technical standards for raw material purification, catalyst life prediction, and spent catalyst recovery, in order to promote methanol reforming hydrogen production from the laboratory to a large-scale green hydrogen plant.

## Figures and Tables

**Table 1:** Mechanism of action and advantages and disadvantages of different supports.

Support Type	Advantage	Inferior Position	Mechanism of Action
ZnO	Stabilize Cu^+^ species and enhance WGSR activity [27]	It is easy to sinter at high temperatures [27]	The Cu–O–Zn interface site was formed to optimize the Cu^+^/Cu^0^ dynamic equilibrium [27]
CeO_2_	High oxygen vacancy concentration, inhibition of carbon deposition [28]	The reduced state has low mechanical strength [28]	Oxygen vacancy promotion · OH migration, accelerating CO oxidation [28]
Al_2_O_3_	High specific surface area, low cost [29]	The strong acidic site exacerbates the carbon deposit [29]	B acid position promotes methanol dehydration to form DME [29]
SiO_2_	The limiting effect inhibits sintering [30]	Weak metal–support interactions [30]	Mesoporous restriction of Cu particle migration [30]
zeolite	The molecular sieve effect blocks poisons [31]	Microporous diffusion limitation [31]	The pore confines Cu particles, which physically block small molecule poisons such as H_2_S [31]

**Table 2:** Multivariate collaborative optimization of nanocomposites.

Tactics	Specific Methods	Effects/Benefits
Alloying strategy	Cu-Ni and Cu-Co bimetallic nanoparticles were introduced to regulate the d-band center through electronic synergy.	Inhibition of CO adsorption, carbon deposition rate reduced by 50%. The H_2_ yield was increased to 3.5 mol/(g_cat·min), and the CO selectivity was <0.5%.
Co-catalyst doping	Doped with ZnO and CeO_2_ nanoparticles (particle size 5–8 nm) to form a strong Cu-ZnO/CeO_2_ interface interaction.	Stabilize Cu^+^ species, reduce methanol dehydrogenation barrier by 0.3 eV. The CO oxidation rate was increased by 12%, and the CO selectivity was <0.3%.
Multi-level pore carrier design	MOF-derived hierarchical pore carbon (mesoporous 3–5 nm, macroporous 50–100 nm) or wood template oriented macroporous structure.	The methanol diffusion flux increased to 5.2 × 10^−6^ mol/(m^2^·s). After 500 h, the Cu particle size only increased by 0.8 nm, and the carbon deposition rate was 0.02 g/(g_cat·h).
Theoretical exploration and model support	DFT calculates the charge transfer at the Cu–ZnO interface. MD simulated the mesoporous confinement effect; machine learning filters the quaternary system.	It was revealed that the H_2_O dissociation barrier decreased from 1.4 eV to 0.9 eV. The ZnO/MgO molar ratio (2:1) was predicted to optimize the carbon deposition inhibition to 0.03 g/(g_cat·h).

**Table 3:** Green nanosynthesis and intelligent regeneration technology.

Technology	Specific Methods	Effects/Benefits
Green synthesis technology	Biomass carrier (rice husk ash mesoporous SiO_2_) [86]	88% dispersion, 70% cost reduction [86]
Intelligent regeneration technology	Temperature-sensitive polymer PNIPAM coating [88]	The start–stop stability is increased by three times [88]
Innovation in the recycling process	Weak acid leaching (0.5 M citric acid) [89]	Activity recovery rate 92% [89]

**Table 4:** Catalyst summary table.

Catalyst System	Preparation Method	Conditions (°C/GHSV)	H_2_ Yield (mol/g·min)	CO Selectivity (%)	Stability (h)
Cu/ZnO/Al_2_O_3_	Co-precipitation	250 /5000 h^−1^	3.0	0.8	500
Cu/ZSM-5	Ion exchange	280 /8000 h^−1^	2.8	1.2	400
CuSiO_2_ (core-shell)	Microemulsion	300 /6000 h^−1^	3.2	0.5	1200

## References

[1] Yan H., Xu Y., Li H., Li W., Liu Y., Wang L. (2015). Study on the activity of catalyst for hydrogen production by copper-based methanol water vapor reforming. Shandong Chem. Ind..

[2] Yan H., Li H., Li W., Xu Y., Liu Y. (2016). Effect of Different Alumina on Catalyst Activity of Copper-based Methanol Water Vapor Reforming for Hydrogen Production. Sichuan Chem. Ind..

[3] Chen M., Mei Z., Chen K., Luo Y. (2017). Research progress on hydrogen production catalyzed by methanol steam reforming with Cu-based catalysts. Petrochem. Technol..

[4] Bai X., Liu C., Wu F., Fan C., Lan W. (2020). Research and application progress of methanol hydrogen production technology. Guangzhou Chem. Ind..

[5] Sun X., Sha Q., Wang C., Zhou D. (2021). Research Progress on Copper-based Catalysts for Methanol Reforming to Hydrogen. CIESC J..

[6] Wang F., Guan D., Dou W. (2022). Research progress on the intensification of methanol steam reforming process for hydrogen production. China Sci. Technol. Pap. Online.

[7] Liu C., Hu X. (2022). Theoretical study on CO_2_ hydrogenation to formic acid and design of high-efficiency iron-based catalyst. Mol. Catal..

[8] Han X., Zhong H., Li J., Song R., Pan L., Tang H. (2022). Research Progress on Cu-based Catalysts for Methanol Vapor Reforming to Hydrogen with Different Carriers. China Biogas.

[9] Zhu S., Shen Y., Hu T., Lu L. (2023). Study on Preparation of Chalcopyrite by Copper-based Catalyst for Methanol Water Vapor Reforming for Hydrogen Production. J. Wuhan Univ. Sci. Technol..

[10] Ju H. (2022). Design and analysis of methanol reforming process for hydrogen production. Guangdong Chem. Ind..

[11] Zhang Z., Jing X., Xu H., Li Z., Yan W. (2023). Research Progress on Design and Reaction Mechanism of Copper-based Catalysts for Electrocatalytic Carbon Dioxide Reduction. Chin. J. Anal. Chem..

[12] Zhang M., Liu Z., Yan Y., Liu D., Xu G., An Y., Zou Y., Yu Y., Francisco J.S., He H. (2025). Optimizing selectivity via steering dominant reaction mechanisms in steam reforming of methanol for hydrogen production. Nat. Commun..

[13] Purnama H. (2003). Catalytic Study of Copper Based Catalysts for Steam Reforming of Methanol. Ph.D. Thesis.

[14] Mortazavi-Manesh A., Safari N., Bahadoran F., Khani Y. (2023). Synthesis, characterization, and methanol steam reforming performance of Cu/perovskite-structured catalysts. Heliyon.

[15] Huo K., Wang Y., Wu M. (2023). Research Progress on Cu-based Catalysts for CO_2_ Hydrogenation to Methanol. Low Carbon Chem. Chem. Eng..

[16] Jia C., Zhang Z., Chen H., Wang Y., Xu G., Chen H. (2023). Zero-carbon new energy absorption system based on hydrogen production by water electrolysis and methanol synthesis/reforming. Mod. Chem. Ind..

[17] Cong X., Zhang Z., Feng Y., Qiu H., Huang C., Tang M., Zhao L. (2023). Effect of La Doping on Hydrogen Production Performance by Methanol Water Vapor Reforming of CuO/CeO_2_ Nanorods. Anhui Chem. Ind..

[18] Shi Y., Lin G., Sun X., Jiang W., Qiao D., Yan B. (2023). Research progress on the active sites of copper-based catalysts in the process of carbon dioxide hydrogenation to methanol. Chem. Ind. Eng. Prog..

[19] Xian J., Gao W., Xu Y., Zhou J., Chen Z., Na W. (2023). Study on Improvement Strategy of Copper Valence Distribution on CO_2_ Hydrogenation to Methanol Catalyst. Mod. Chem. Ind..

[20] Chen F., Zhong Z., Qi R. (2024). Research Progress on Electroreduction of Carbon Dioxide to Formic Acid by Copper-based Catalysts. Chem. Ind. Eng. Prog..

[21] Chen Z., Zhuang Y., Du W., Yin D., Deng Y. (2023). A brief analysis on the R&D and application prospect of CO_2_ hydrogenation to methanol. Dongfang Electr. Rev..

[22] Li H., Xiao Y., Xiao J., Fan K., Li B., Li X., Wang L., Xiao F. (2023). Gallium-modified copper-based hydrophobic catalyst for selective hydrogenation of CO_2_ to dimethyl ether. Chin. J. Catal..

[23] Liu J., Yu Y., Yuan H. (2024). Research Progress on Performance Regulation of Copper-based Catalysts for Methyl Acetate Hydrogenation to Ethanol. Low Carbon Chem. Chem. Eng..

[24] Lytkina A.A., Orekhova N.V., Ermilova M.M., Yaroslavtsev A. (2018). The influence of the support composition and structure (MXZr1-XO2-δ) of bimetallic catalysts on the activity in methanol steam reforming. Int. J. Hydrogen Energy.

[25] Blanco H., Palacio L.A., Rodrigues V., Faro A. (2024). Cu–Mn–Al based catalyst for the direct syngas to dimethyl ether conversion. React. Kinet. Mech. Catal..

[26] Tedeeva M.A., Kustov A.L., Batkin A.M., Garifullina C., Zalyatdinov A.A., Yang D., Dai Y., Yang Y., Kustov L.M. (2024). Catalytic systems for hydrogenation of CO_2_ to methanol. Mol. Catal..

[27] Jitrwung R., Krekkeitsakul K., Teerananont N., Thongyindee P., Patthaveekongka W., Areeprasert C. (2025). Utilization of CO_2_ and recycling of methanol Residue from the refining process for production of Bio-Methanol. Carbon Resour. Convers..

[28] Hu L., Dai C., Chen L., Zhu Y., Hao Y., Zhang Q., Gu L., Feng X., Yuan S., Wang L. (2021). Metal-triazolate-framework-derived FeN4Cl1 single-atom catalysts with hierarchical porosity for the oxygen reduction reaction. Angew. Chem. Int. Ed..

[29] Chen L.H., Sun M.H., Wang Z., Yang W., Xie Z., Su B.-L. (2020). Hierarchically structured zeolites: From design to application. Chem. Rev..

[30] Tonekaboni M.F., Rezaei M., Alavi S.M., Akbari E. (2025). Synthesis and evaluation of Cu-based catalysts employed in the medium temperature water gas shift reaction. Int. J. Hydrogen Energy.

[31] Li J., Lin W., Lu M., Liao J., Hu C., Wang T. (2024). High density ultra-small Cu nanoparticles with abundant oxygen vacancies for efficient hydrogen evolution from MeOH/H_2_O. J. Catal..

[32] Yu X., Yang J., Tan J., Du J., Ru Y. (2024). Current status and development trend of methanol synthesis technology. Energy Chem. Ind..

[33] Guo J., Cao L., Wang Z. (2024). Research Progress on Methanol Water Steam Reforming for Hydrogen Production. Energy Conserv..

[34] Huang M., Bo Q., Li J., Qiao J., Yuan S., Zhang B., Chen H., Jiang Y. (2024). Effect of Al_2_O_3_ Precursor on the Performance of Cu/ZnO/Al_2_O_3_ in Catalyzing Methanol Reforming for Hydrogen Production. J. Fuel Chem. Technol..

[35] Lv Y., Zhang Y., Wang R., Wu Q., Shi D., Chen K., Li H. (2024). Research Progress on Copper-based Catalysts for Reverse Water Vapor Shift Reaction. Ind. Catal..

[36] Zou J., Zhang J., Wu X., Wei C., Fang S., Wang Y. (2024). Comprehensive Experiment on Nitrate Synthesis of Ammonia Based on Electrocatalytic Reduction: Preparation Characterization, Performance Exploration and Application Design of Copper-based Catalyst. Univ. Chem..

[37] Zhu G., Ge Q., Fu M. (2025). Durability evaluation and life prediction method of methanol reforming catalyst for hydrogen production. Chem. Ind. Eng. Prog..

[38] Mateos-Pedrero C., Silva H., Tanaka D.A.P., Liguori S., Iulianelli A., Basile A., Mendes A. (2015). CuO/ZnO catalysts for methanol steam reforming: The role of the support polarity ratio and surface area. Appl. Catal. B Environ..

[39] Zhang L., Pan L., Ni C., Sun T., Zhao S., Wang S., Wang A., Hu Y. (2013). CeO_2_–ZrO_2_-promoted CuO/ZnO catalyst for methanol steam reforming. Int. J. Hydrogen Energy.

[40] Fornari A.C., Pimenta J.L.C.W., dos Santos O.A.A., Jorge L.M.d.M. (2021). Statistical optimization of the composition of CuO–ZnO/Al_2_O_3_ catalysts for methanol steam reforming. Braz. J. Chem. Eng..

[41] Rostami M., Farajollahi A.H., Amirkhani R., Farshchi M.E. (2023). A review study on methanol steam reforming catalysts: Evaluation of the catalytic performance, characterizations, and operational parameters. AIP Adv..

[42] Hafezi-Bakhtiari J., Bazyari A., Rezaei M., Akbari E., Babaei E. (2024). Optimizing bimetallic and multimetallic Cu-based catalysts by promoters for enhanced reverse Water-Gas Shift reaction: Insights and stability assessments. J. CO2 Util..

[43] Pathak R., Punetha V.D., Bhatt S., Punetha M. (2024). A review on copper-based nanoparticles as a catalyst: Synthesis and applications in coupling reactions. J. Mater. Sci..

[44] Yesupatham M.S., Honnappa B., Agamendran N., Kumar S.Y., Chellasamy G., Govindaraju S., Yun K., Selvam N.C.S., Maruthapillai A., Li W. (2024). Recent Developments in Copper-Based Catalysts for Enhanced Electrochemical CO_2_ Reduction. Adv. Sustain. Syst..

[45] Zheng M., Zhang J., Wang P., Jin H., Zheng Y., Qiao S. (2024). Recent advances in electrocatalytic hydrogenation reactions on copper-based catalysts. Adv. Mater..

[46] Li X., You J., Li J., Wang Z., Zhao Y., Xu J., Duan M., Zhang H. (2024). Progress of Copper-based Nanocatalysts in Advanced Oxidation Degraded Organic Pollutants. ChemCatChem.

[47] Nikam R.R., Manikanta P., Patil K.N., Mounesh, Kainthla I., Patil S.A., Nagaraja B.M. (2025). State-of-the-art for the development of Cu-based heterogeneous catalysts for efficient utilization of furfural to value chemicals via liquid-phase and gas-phase reactions. Catal. Rev..

[48] Luo C., Liu X., Yang K., Xu J., Zhu Z., Tang Z., Shen S., Fan J., Luo D., Alshammari N.A.H. (2024). Coordination structure engineering of Cu-based electrocatalysts for electrocatalytic water splitting. Coord. Chem. Rev..

[49] Zhong Y., Sun Z., Xia B.Y., Su Y. (2025). Structural Reconstruction of Copper-Based Catalysts in CO_2_ Electroreduction Reaction: A Comprehensive Review. Chem. A Eur. J..

[50] Bonthula S., Bonthula S.R., Pothu R., Srivastava R.K., Boddula R., Radwan A.B., Al-Qahtani N. (2023). Recent advances in copper-based materials for sustainable environmental applications. Sustain. Chem..

[51] Han J., Ma J., Zhou J., Chen X., Wan Z., Zhao Y. (2023). Insight into the effect of surface coverage of carbon support on selective CO_2_ electroreduction to C_2_H_4_ over copper-based catalyst. Appl. Surf. Sci..

[52] Qi X., Wang Y., Liu C., Liu Q. (2023). The challenges and comprehensive evolution of Cu-based zeolite catalysts for SCR systems in diesel vehicles: A review. Catal. Surv. Asia.

[53] Li M., Hu Y., Wu T., Sumboja A., Geng D. (2023). How to enhance the C2 products selectivity of copper-based catalysts towards electrochemical CO_2_ reduction?—A review. Mater. Today.

[54] Zhao M., Yin W., Xiao J., Dong J., Deng J., Li L., Li X., Li X., Peng B., Dong H. (2025). Peracetic acid activation by chitosan-derived nitrogen-doped carbon spheres loaded with zero-valent copper for efficient sulfamethazine degradation in groundwater. Sep. Purif. Technol..

[55] Huang J., Ning S., Luo B., Wang Z., Deng W., Zhao B., Su Y. (2024). Copper-based catalysts supported on novel Metal-Organic Framework MIL-125 (Ti) for selective catalytic reduction of NO with CO. Fuel.

[56] Wang F., Lu Z., Guo H., Hao G., Jiang W., Liu G. (2024). Copper-based catalysts for CO_2_ electroreduction to C2/2+ products: Advance and perspective. Coord. Chem. Rev..

[57] Kute A.D., Gaikwad R.P., Warkad I.R., Gawande M.B. (2022). A review on the synthesis and applications of sustainable copper-based nanomaterials. Green Chem..

[58] Sayyahi S., Saghanezhad S.J. (2019). Copper-based bulk and nano-catalysts for the one-pot propargylamine synthesis. Mini-Rev. Org. Chem..

[59] Gordeychuk D.I., Sorokoumov V.N., Mikhaylov V.N., Panov M.S., Khairullina E.M., Melnik M.V., Kochemirovsky V.A., Balova I.A. (2020). Copper-based nanocatalysts produced via laser-induced ex situ generation for homo-and cross-coupling reactions. Chem. Eng. Sci..

[60] Wang D., Zhou P., Tang J., Yang L., Jie D. (2024). Numerical simulation of catalytic reaction kinetics in a sine wave tubular methanol steam reforming reactor. Int. J. Hydrogen Energy.

[61] Zaza L., Rossi K., Buonsanti R. (2022). Well-defined copper-based nanocatalysts for selective electrochemical reduction of CO_2_ to C2 products. ACS Energy Lett..

[62] Zuo W., Fan Z., Chen L., Liu J., Wan Z., Xiao Z., Chen W., Wu L., Chen D., Zhu X. (2022). Copper-based theranostic nanocatalysts for synergetic photothermal-chemodynamic therapy. Acta Biomater..

[63] Nasrollahzadeh M., Ghorbannezhad F., Issaabadi Z., Sajadi S.M. (2019). Recent developments in the biosynthesis of Cu-based recyclable nanocatalysts using plant extracts and their application in the chemical reactions. Chem. Rec..

[64] Jiang Y. (2024). Current status and development trend of CO_2_ hydrogenation to methanol. Energy Chem. Ind..

[65] Sampatkumar H.G., Gundakanal S.S., Gowda B., Doddamani S.V., Sasidhar B.S., Bugarin A., Patil S.A. (2025). Catalyst to cure: Applications of a new copper-based nanocatalyst in organic synthesis and cancer treatment. Chem. Commun..

[66] Maleki M.H., Shirani M.A., Dinari M. (2022). Facile synthesis of green and efficient copper-based magnetically recoverable nanocatalyst for the reduction of nitrophenol derivatives. J. Mol. Liq..

[67] Zheng Y., Li D., Min Y., Li Y., Huang H. (2024). Design strategy of copper-based catalyst for electrocatalytic carbon dioxide reduction. Clean Coal Technol..

[68] Dong J., Shan M., Wang H. (2025). Au-CuO/Cu_2_O tandem catalytic enhanced electrocatalytic reduction of CO_2_ to ethanol. Chem. Ind. Eng. Prog..

[69] Sanati-Tirgan P., Eshghi H., Mohammadinezhad A. (2024). Synthesis and catalytic applications of highly stable copper-based metal–organic framework as an efficient heterogeneous nanocatalyst for solvent-free Suzuki–Miyaura cross-coupling reaction. Appl. Organomet. Chem..

[70] He Q., Li Z., Wang Q., Zhang J., Liu C. (2024). Research Progress on Hydrophobic Modification of Cu-based Catalysts for Carbon Dioxide Hydrogenation to Methanol. Petrochem. Technol. Appl..

[71] Priya S.C., Vijayalakshmi S., Raghavendra S.G., Yıldızhan S., Ranjitha J. (2023). A critical review on efficient photocatalytic degradation of organic compounds using copper-based nanoparticles. Mater. Today Proc..

[72] Li S., Yan Q., Jia X., Ning N., Ge P. (2024). Hydrogen production performance of Methanol water vapor reforming monolithic catalyst with CuO/ZnO/Al_2_O_3_ stainless steel fiber felt structure. Chem. React. Eng. Technol..

[73] Lin T., Cheng J., Zhang B., Fan J., Gao M., Zheng J., Chao Z., Li G., Ma X., Du H. (2024). Dehydrogenation of propylene glycol methyl ether to methoxyacetone on copper-based catalyst. Ind. Catal..

[74] Sharma K., Khatik N., Khandelwal A.R., Meena R., Bhadauria S., Singh M., Sachdeva H. (2023). Ecofriendly Synthesis of DHPMs using Copper-based Nano catalysts and Evaluation of Antibacterial Activity. Croat. Chem. Acta.

[75] Cao K.L.A., Kautsar D.B., Kume K., Le Cao K.A., Septiani E.L., Hirano T., Tsunoji N., Matsukata M., Ogi T. (2025). Preparation of Hierarchical Porous Zeolite Particles with Multiscale Pore Architectures through a Template-Assisted Spray Process for Enhanced Toluene Adsorption Rate. ACS Appl. Mater. Interfaces.

[76] Bagherzadeh S.B., Haghighi M. (2020). Texture-evolution of copper-based nanocatalyst via hybrid glow discharge plasma-oxalate-precipitation method for hydrogen production from CH_3_OH/H_2_O mixture. Appl. Catal. A Gen..

[77] Cruz-Navarro J.A., Hernández-García F., Sánchez-Mora A.T., Moreno-Narváez M.E., Reyes-Márquez V., Colorado-Peralta R., Morales-Morales D. (2024). Copper-Based Metal–Organic Frameworks Applied as Electrocatalysts for the Electroreduction of Carbon Dioxide (CO_2_ER) to Methane: A Review. Methane.

[78] Kembo J.P.N., Wang J., Luo N., Gao F., Yi H., Zhao S., Zhou Y., Tang X. (2023). A review of catalytic oxidation of carbon monoxide over different catalysts with an emphasis on hopcalite catalysts. New J. Chem..

[79] Dehkordi S.S.S., Jafari A.A., Albadi J., Samimi H.A. (2023). Mesoporous epoxidized soybean oil-supported copper-based magnetic nanocatalyst and amberlite-supported azide as a green and efficient catalytic system for 1, 2, 3-triazole synthesis. Mol. Divers..

[80] Nwosu U., Siahrostami S. (2023). Copper-based metal–organic frameworks for CO_2_ reduction: Selectivity trends, design paradigms, and perspectives. Catal. Sci. Technol..

[81] Elmehrath S., Ahsan K., Munawar N., Alzamly A., Nguyen H.L., Greish Y. (2024). Antibacterial efficacy of copper-based metal–organic frameworks against Escherichia coli and Lactobacillus. RSC Adv..

[82] Hu Y., Xu K., Yang H., Zhang F., Yang Z., Dong Z. (2025). Copper-based catalyst for CO_2_ electrocatalysis to ethylene production. Prog. Chem..

[83] Ebrahimiasl H., Azarifar D. (2020). Copper-based Schiff Base Complex Immobilized on Core-shell Fe_3_O_4_@ SiO_2_ as a magnetically recyclable and highly efficient nanocatalyst for green synthesis of 2-amino-4H-chromene derivatives. Appl. Organomet. Chem..

[84] Zhang Y., Yin Y., Jia Z., Jiang M., Gong Z. (2025). Study on the catalytic properties of copper-based composite oxides for CO oxidation. Rare Met. Cem. Carbide.

[85] Ebrahimi P., Kumar A., Khraisheh M. (2020). A review of recent advances in water-gas shift catalysis for hydrogen production. Emergent Mater..

[86] Soni A., Kumar P., Tomar V., Joshi R.K., Nemiwal M. (2022). Recent advances in copper oxide nanocatalyzed CC cross-coupling transformations. Results Chem..

[87] Kumar S., Muhammad R., Amhamed A., Oh H. (2025). Unveiling the potential of ingenious copper-based metal-organic frameworks in gas storage and separation. Coord. Chem. Rev..

[88] Haso H.W., Dubale A.A., Chimdesa M.A., Atlabachew M. (2022). High performance copper based metal organic framework for removal of heavy metals from wastewater. Front. Mater..

[89] Akbarpour T., Khazaei A., Yousefi Seyf J., Sarmasti N. (2021). Synthesis of 1-aminoalkyl-2-naphthols derivatives using an engineered copper-based nanomagnetic catalyst (Fe_3_O_4_@CQD@Si (OEt)(CH_2_) _3_NH@CC@N_3_@phenylacetylene@Cu). Appl. Organomet. Chem..

[90] Abouri M., Elouardi M., Mabrouki J., El Belghiti M.A., El Hamidi A. (2025). Copper and Copper-Based Nanoparticles for Water Treatment. Technical Innovation and Modeling in the Biological Sciences.

[91] Gaete J., Valdebenito G., Moglia I., Morales-Verdejo C., Aguirre P., Fernandes J.A., Abarca G. (2025). Copper-based magnetic nanocatalysts for the catalytic transfer hydrogenation of biomass-derived furfural. Appl. Surf. Sci..

[92] Zhang C., Ying Y., Jia Y., Zhao Z., Li Y., Shi J. (2024). Research Progress on Failure Mechanism of Copper-based Catalysts in Electrocatalytic CO2 Reduction. Copp. Eng..

[93] Qiu Y., Ye H., Chen J., Liu D., Gao Z., Zhang Y. (2024). Research Progress on Integral Catalysts Based on Reforming Hydrogen Production Technology. Contemp. Chem. Ind..

[94] Duan J., Yin M., Kang M., Sun R., Xu Y., Yan H. (2024). Application of SiO_2_@TiO_2_ catalyst support in hydrogen production by methanol reforming. J. Southwest Univ. Sci. Technol..

[95] Bouzayani B., Lomba-Fernández B., Fdez-Sanromán A., Elaoud S.C., Sanromán M.Á. (2024). Advancements in Copper-Based Catalysts for Efficient Generation of Reactive Oxygen Species from Peroxymonosulfate. Appl. Sci..

[96] Ahmed S., Abdullah I., Krisnandi Y.K. (2025). Harnessing Copper’s Potential: A Review of Cu-Based Catalysts for Glycerol Conversion. Bull. Chem. React. Eng. Catal..

[97] Kouzehli A., Kazemeini M., Moghaddam A.A. (2024). A robust kinetic modeling of CO_2_ hydrogenation to methanol over an industrial copper–zinc oxide catalyst based on a single-active site mechanism. Int. J. Hydrogen Energy.

[98] Huang H., Na W., Gao W., Huang Z. (2024). Performance of CO_2_ Hydrogenation to Methanol from La-Cu/ZnO/ZrO_2_ Catalyst. J. Funct. Mater..

[99] Yan B., Cao J., Sun X., Li B. (2024). Research Progress on CO_2_-Catalyzed Hydrogenation of Metal-Organic Framework-Supported Metal Catalysts. Sci. China Chem..

[100] Sun G., Yao Y., Chen Y., Zhao K., Cai J., Fu C., Lin J. (2024). Research progress on electrocatalytic carbon dioxide reduction by copper-based catalysts. J. Fujian Norm. Univ. Technol..

[101] Liu Y., Yang X., Ruan J., Wu M., Cao Y. (2024). Research Progress on Electrocatalytic CO_2_ Reduction to Produce C+2 Products. Mod. Chem. Ind..

[102] Zhang Z., Baker R., Liu Z., Zhou M., Deng G., Wei W., Mao L., Li H., Jiang Z. (2024). Research progress on carbon dioxide conversion of copper-based photocatalysts. Acta Phys.-Chim. Sin..

[103] Wang F., Guan D., Zhang X., Wu C., Wang G. (2024). Thermodynamic analysis of hydrogen production by methanol water vapor reforming under induction heating. J. Contemp. Chem. Ind. Eng. Res..

[104] Wu Z., Liu D., Yin H. (2025). Research Progress on Methanol Liquid Phase Reform Catalysts for Hydrogen Production. Chin. Chem. Bull..

[105] Liu L., Liu R., Liu Z., Liu X., Zheng H., Zeng L., Xin F. (2025). Study on the performance of chemical chain methanol oxidation steam reforming process. Chem. React. Eng. Technol..

[106] Du Z., Liu B. (2025). Catalytic performance of Cu/SiO_2_ catalyst for hydrogen production by methanol steam reforming. Energy Chem. Ind..

[107] Jiang Y., Deng L., Liu Y., Liu H. (2025). Application of copper-based catalysts in electrocatalytic CO_2_ to C_2+_ products. Mod. Chem. Ind..

[108] Zhao T., Gao T., Yang Y., Xu D., Han T., Deng B., Milgen B. (2025). Research status and prospect of CO_2_ hydrogenation to methanol on copper-based catalysts. Clean Coal Technol..

[109] Kumar A., Singh R., Sinha A.S.K. (2019). Catalyst modification strategies to enhance the catalyst activity and stability during steam reforming of acetic acid for hydrogen production. Int. J. Hydrogen Energy.

[110] Tommasi M., Ceriotti D., Gramegna A., Degerli S.N., Ramis G., Rossetti I. (2024). Oxidative Steam Reforming of Methanol over Cu-Based Catalysts. Catalysts.

[111] Nippes R.P., Macruz P.D., Domingues Gomes A., de Souza M., Ferreira B.R., Rizzo-Domingues R.C.P., Ramos L.P. (2024). Effect of Support on Steam Reforming of Ethanol for H2 Production with Copper-Based Catalysts. Processes.

[112] Słowik G., Rotko M., Ryczkowski J., Greluk M. (2024). Hydrogen production from methanol steam reforming over Fe-modified Cu/CeO_2_ catalysts. Molecules.

[113] Barberis L., Versteeg C.I., Meeldijk J.D., Stewart J.A., Vandegehuchte B.D., de Jongh P.E. (2024). K and Na promotion enables high-pressure low-temperature reverse water gas shift over copper-based catalysts. ACS Catal..

[114] Farooq M.S., Baig A., Wei Y., Liu H., Zeng Z., Shi Z. (2024). A comprehensive analysis of a compact-sized methanol cracking unit for hydrogen production. Int. J. Hydrogen Energy.

[115] Achomo M.A., Kumar A., Muthukumar P., Peela N.R. (2024). Experimental studies on hydrogen production from steam reforming of methanol integrated with metal hydride-based hydrogen purification system. Int. J. Hydrogen Energy.

[116] Qasmi C., Mochel R., Gautier V., Champon I., Thomas S., Chappaz A., Anne-Cécile R. (2024). GBL/BDO pair as a bio-based liquid organic carrier: Kinetic modeling of liquid phase hydrogenation and dehydrogenation over copper-based catalyst. Int. J. Hydrogen Energy.

[117] Mdlovu N.V., Lin K.S., Yeh H.P., François M., Hussain A., Badshah S.M. (2025). Autothermal reforming of methanol in a microreactor using porous alumina supported CuO/ZnO with CeO_2_ sol catalysts washcoat. J. Energy Inst..

[118] Zonouz H.V., Barzegari F., Rezaei M. (2024). Development of CuO/ZnO/Al_2_O_3_-hydrotalcite− based catalysts for middle temperature water gas shift reaction: Impact of calcination temperature and residual carbonates. Fuel.

[119] Chelvam K., Hanafiah M.M., Alkhatib I.I.I., Ali S.M., Vega L.F. (2025). Life cycle assessment on the role of H_2_S-based hydrogen via H_2_S-methane reforming for the production of sustainable fuels. Sci. Total Environ..

[120] Gwon K., Lee S., Kim Y., Choi J., Kim S., Kim S.-J., Hong H.J., Hwang Y., Mori M., Lee D.N. (2023). Construction of a bioactive copper-based metal organic framework-embedded dual-crosslinked alginate hydrogel for antimicrobial applications. Int. J. Biol. Macromol..

[121] Abdelhameed R.M., El-Shahat M., Abdel-Gawad H., Hegazi B. (2023). Efficient phenolic compounds adsorption by immobilization of copper-based metal-organic framework anchored polyacrylonitrile/chitosan beads. Int. J. Biol. Macromol..

[122] Qian Y., Wang C., Xu R., Wang J., Chen Q., Zhu Z., Hu Q., Shen Q., Shen J.-W. (2025). Copper-based metal–organic frameworks for antitumor application. J. Nanobiotechnol..

[123] Li S., Huang L., Jia B., Feng X., Cao Y., Chen Y., Bin Y. (2024). Effect and mechanism of inorganic anions on the adsorption of Cd^2+^ on two-dimensional copper-based metal–organic framework. Inorg. Chem. Commun..

[124] Morales-Cámara S., Toral V., Vitorica-Yrezabal I.J., Rivadeneyra A., Pereira L., Rojas S., Romero F.J. (2024). Simple fabrication of laser-induced graphene functionalized with a copper-based metal–organic framework and its application in solid-state supercapacitors. J. Mater. Chem. C.

[125] Karimi-Nazarabad M., Azizi-Toupkanloo H., Sajjadizadeh H.S., Goharshadi E.K., Jorabchi M.N. (2024). Enhanced oxygen evolution of a new copper-based metal-organic framework through the construction of a heterogeneous structure with bismuth oxyiodide. Electrochim. Acta.

[126] Sun M., Hanif A., Wang T., Gu Q., Shang J. (2023). Ambient temperature NO_2_ removal by reversible NO_2_ adsorption on copper-based metal-organic frameworks (MOFs)-derived nanoporous adsorbents. Sep. Purif. Technol..

[127] Jeon I.J., Lee J.S., Baek K.W., Kim C.-H., Gong J.-H., Jang W.-J., Cho J.S., Shim J.-O. (2025). Highly dispersed copper-based nanocomposite synthesis via spray pyrolysis: Towards waste-to-hydrogen production through the water-gas shift reaction. J. Mater. Chem. A.

[128] Islam S.M.S., Yasmeen R., Verma G., Tekarli S.M., Nesterov V.N., Ma S., Omary M.A. (2024). A Copper-Based Metal–Organic Framework for Selective Separation of C2 Hydrocarbons from Methane at Ambient Conditions: Experiment and Simulation. Inorg. Chem..

[129] Dawidowicz R., Patrascu M. (2024). Methanol compared to other fuels for on-board hydrogen production in thermally balanced membrane reactors with internal recycle. Chem. Eng. Process.-Process Intensif..

[130] Kadeer K., Jiang Y., Huang Y., Lin Y., Jin R., Yin C., Guo F., Ichikawa T., Li X., Zheng J. (2025). Hydrogen generation from coupled methanol steam reforming with metal hydride hydrolysis: Effects of metal catalysts and hydrides. Int. J. Hydrogen Energy.

[131] Caldeira A.C.R., Alves H.O., da Silva A.H.M., Gomes J.F. (2024). Influence of water steam and copper oxidation state on the CO_2_ hydrogenation to ethanol over copper catalysts. Catal. Today.

[132] Ismail R., Saad M.A.H., Al-Marri M.J., Sardar A., Mohamed A.T., El-Naas M., Soliman A.M., Benamor A. (2024). Synthesis and evaluation of novel Cu-based adsorbent-containing catalysts for CO_2_ hydrogenation to methanol and value-added products. J. Environ. Chem. Eng..

